# γ-Tubulin 2 Nucleates Microtubules and Is Downregulated in Mouse Early Embryogenesis

**DOI:** 10.1371/journal.pone.0029919

**Published:** 2012-01-03

**Authors:** Stanislav Vinopal, Markéta Černohorská, Vadym Sulimenko, Tetyana Sulimenko, Věra Vosecká, Matyáš Flemr, Eduarda Dráberová, Pavel Dráber

**Affiliations:** 1 Department of Biology of Cytoskeleton, Institute of Molecular Genetics, Academy of Sciences of the Czech Republic, Prague, Czech Republic; 2 Department of Epigenetic Regulations, Institute of Molecular Genetics, Academy of Sciences of the Czech Republic, Prague, Czech Republic; Institut de Génétique et Développement de Rennes, France

## Abstract

γ-Tubulin is the key protein for microtubule nucleation. Duplication of the γ-tubulin gene occurred several times during evolution, and in mammals γ-tubulin genes encode proteins which share ∼97% sequence identity. Previous analysis of *Tubg1* and *Tubg2* knock-out mice has suggested that γ-tubulins are not functionally equivalent. *Tubg1* knock-out mice died at the blastocyst stage, whereas *Tubg2* knock-out mice developed normally and were fertile. It was proposed that γ-tubulin 1 represents ubiquitous γ-tubulin, while γ-tubulin 2 may have some specific functions and cannot substitute for γ-tubulin 1 deficiency in blastocysts. The molecular basis of the suggested functional difference between γ-tubulins remains unknown. Here we show that exogenous γ-tubulin 2 is targeted to centrosomes and interacts with γ-tubulin complex proteins 2 and 4. Depletion of γ-tubulin 1 by RNAi in U2OS cells causes impaired microtubule nucleation and metaphase arrest. Wild-type phenotype in γ-tubulin 1-depleted cells is restored by expression of exogenous mouse or human γ-tubulin 2. Further, we show at both mRNA and protein levels using RT-qPCR and 2D-PAGE, respectively, that in contrast to *Tubg1*, the *Tubg2* expression is dramatically reduced in mouse blastocysts. This indicates that γ-tubulin 2 cannot rescue γ-tubulin 1 deficiency in knock-out blastocysts, owing to its very low amount. The combined data suggest that γ-tubulin 2 is able to nucleate microtubules and substitute for γ-tubulin 1. We propose that mammalian γ-tubulins are functionally redundant with respect to the nucleation activity.

## Introduction

γ-Tubulin is a highly conserved member of the tubulin superfamily essential for microtubule nucleation in all eukaryotes [1]–[3]. It assembles together with other proteins, named Gamma-tubulin Complex Proteins (GCPs) in human, into two main γ-Tubulin Complexes (γTuCs): the γ-Tubulin Small Complex (γTuSC) and the γ-Tubulin Ring Complex (γTuRC). The γTuSC, a vital component of microtubule nucleation machinery in all eukaryotes, is composed of two molecules of γ-tubulin and one copy each of GCP2 and GCP3. The γTuRCs are found only in metazoa and consist of seven γTuSCs and additional GCPs, including GCP4-6 [4], [5]. The γTuRC is a ring structure with an arrangement of γ-tubulin molecules that matches the 13-fold symmetry of a microtubule. It serves as a template for microtubule polymerization [6]. It has recently been shown that the budding yeast γTuSCs alone form *in vitro* ring structures similar to γTuRCs [7]; it supports the general template model of microtubule nucleation [6].

γTuCs are concentrated at Microtubule Organizing Centers (MTOCs) such as centrosomes and basal bodies in animals or spindle pole bodies in fungi. They are also found on nuclear membranes in acentrosomal plants and on Golgi membranes, condensed mitotic chromosomes, midbodies and along microtubules in mitotic spindles [8]. We have recently reported nucleolar localization of γ-tubulin [9]. However, the majority of γTuCs exist in cytoplasm in soluble form [10]. In addition to its function in microtubule nucleation, γ-tubulin is also involved in centriole biogenesis [11], [12], regulation of microtubule (+) end dynamics [13]–[15], regulation of the anaphase-promoting complex/cyclosome during interphase in *Aspergillus* [16] or regulation of bipolar spindle assembly in fission yeast [17].

Many organisms including *Arabidopsis* [18], *Paramecium* [19], *Euplotes* [20], *Drosophila* [21] and mammals [22]–[24] possess two genes encoding γ-tubulin. Nevertheless, phylogenetic analyses revealed that γ-tubulin gene duplication in mammals occurred independent of the others [23], [24]. Mammalian γ-tubulin genes are located on the same chromosome in tandem, and their coding sequences share very high sequence similarity (>94% in human)[22]. Although it was initially assumed that γ-tubulin genes are functionally redundant [22], gene knock-out analysis of *Tubg1* and *Tubg2* in mice suggested that they might have different functions [23]. While *Tubg1* was expressed ubiquitously, *Tubg2* was primarily detected in brain and also in blastocysts. *Tubg1^-/-^* embryos stopped their development at the morula/blastocyst stage because of severe mitotic defects. *Tubg2^-/-^* mice developed normally and produced fertile offspring. However, adults exhibited some behavioral changes including abnormalities in circadian rhythm and different reaction to painful stimulations. These findings led to a conclusion that γ-tubulin 1 is the conventional γ-tubulin, whereas γ-tubulin 2, which lacks the capability to rescue the consequences of γ-tubulin 1 deficiency, might have specific function(s) in the brain [23]. Nevertheless, the molecular basis of suggested functional differences between γ-tubulin 1 and γ-tubulin 2 is unknown.

To gain a deeper insight into the potential functional differences of mammalian γ-tubulins, we have examined subcellular distribution of γ-tubulin 2 in cultured cells, its interactions with GCPs, capability to nucleate microtubules and substitute for γ-tubulin 1. We have also analyzed γ-tubulin 2 expression in the course of mouse preimplantation development. Our results indicate that even though γ-tubulins are differentially expressed during mouse early embryogenesis and in adult tissues, they are functionally redundant with respect to their nucleation activity.

## Results

### γ-Tubulin 2 is indistinguishable from γ-tubulin 1 in subcellular localization and interactions with GCPs

**Figure 1:**
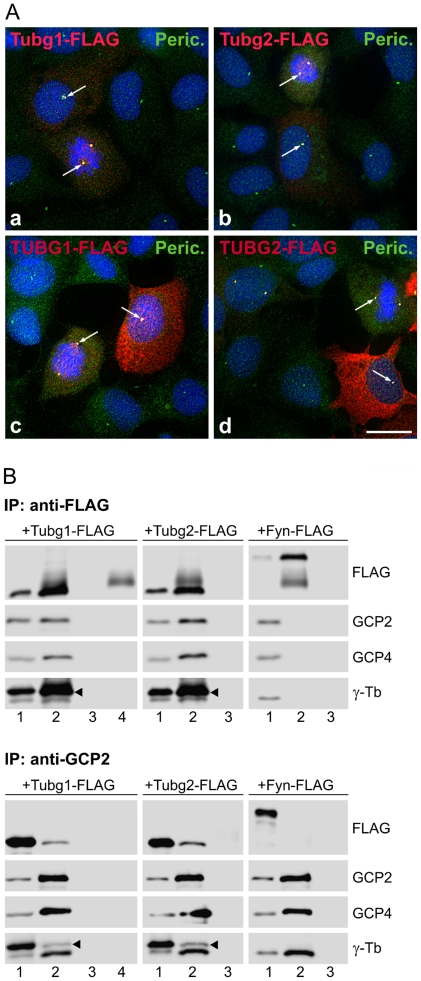
Exogenous γ-tubulin 2 locates to centrosomes and interacts with GCPs. (A) Localization of FLAG-tagged γ-tubulins. Human U2OS cells expressing mouse γ-tubulin 1 (a, Tubg1-FLAG), mouse γ-tubulin 2 (b, Tubg2-FLAG), human γ-tubulin 1 (c, TUBG1-FLAG) and human γ-tubulin 2 (d, TUBG2-FLAG) were stained for FLAG (red) and pericentrin (green). DNA was stained with DAPI (blue). Arrows denote positions of MTOCs where FLAG-tagged γ-tubulins co-localize with pericentrin. Final images were made by maximum intensity projection of 3 deconvolved z-sections spaced at 0.25 µm. Scale bar 10 µm. (B) Coimmunoprecipitation of mouse γ-tubulins with GCP2 and GCP4 proteins. Extracts from HEK cells expressing FLAG-tagged γ-tubulin 1 (Tubg1-FLAG), γ-tubulin 2 (Tubg2-FLAG) or control mouse Fyn (Fyn-FLAG) were immunoprecipitated with antibodies to FLAG or GCP2, and blots were probed with antibodies to FLAG, GCP2, GCP4 and γ-tubulin (γ-Tb). Extracts (1), immunoprecipitated proteins (2), protein A without antibodies incubated with extracts (3), immobilized antibodies not incubated with extracts (4). Arrowheads indicate the positions of exogenous γ-tubulins.

To decide whether or not γ-tubulin 2 differs from γ-tubulin 1 in subcellular localization, we examined U2OS cells expressing FLAG-tagged mouse or human γ-tubulin 2 (Tubg2-FLAG, TUBG2-FLAG) by immunofluorescence microscopy with anti-FLAG antibody. Centrosomes were marked with antibody to pericentrin. U2OS cells expressing FLAG-tagged mouse and human γ-tubulin 1 (Tubg1-FLAG, TUBG1-FLAG) served as controls. As expected, exogenous mouse (Fig. 1A, a) and human (Fig. 1A, c) γ-tubulin 1 localized to the centrosomes in both interphase and mitotic cells. FLAG-tagged γ-tubulin 1 was also found along mitotic spindle and diffusely in cytoplasm. The same staining pattern was detected in cells expressing exogenous mouse (Fig. 1A, b) and human (Fig. 1A, d) γ-tubulin 2. Fully displayed immunofluorescence of Fig. 1A appears in Fig. S1.

Next we checked by coimmunoprecipitation the ability of γ-tubulin 2 to interact with GCP2 (γTuSC marker) and GCP4 (γTuRC marker). FLAG-tagged mouse γ-tubulin 1, γ-tubulin 2 or Fyn kinase (negative control) were immunoprecipitated from HEK 293FT cells with rabbit anti-FLAG antibody. Immunoblot analysis revealed that both FLAG-tagged γ-tubulins interacted with GCP2 and GCP4, yet no coimmunoprecipitation was observed in case of FLAG-tagged Fyn kinase (Fig. 1B, upper panel). Negative control rabbit antibody failed to coimmunoprecipitate GCP proteins (not shown). In addition, the reciprocal precipitation with antibody to GCP2 (IgG2b), confirmed the interaction of FLAG-tagged γ-tubulins with GCP2 (Fig. 1B, lower panel). Again, negative control antibody (IgG2b) did not coimmunoprecipitate FLAG-tagged γ-tubulins (not shown). We obtained the same results when lysates from HEK 293FT cells expressing FLAG-tagged human γ-tubulin 1 and γ-tubulin 2 were used for immunoprecipitation with anti-FLAG and anti-GCP2 antibodies (Fig. S2). Altogether the data indicate that mammalian γ-tubulin 2 is indiscernible from γ-tubulin 1 as far as the subcellular distribution and interactions with components of small and large γ-tubulin complexes are concerned.

### γ-Tubulin 2 rescues mitotic progression in γ-tubulin 1-depleted cells

**Figure 2:**
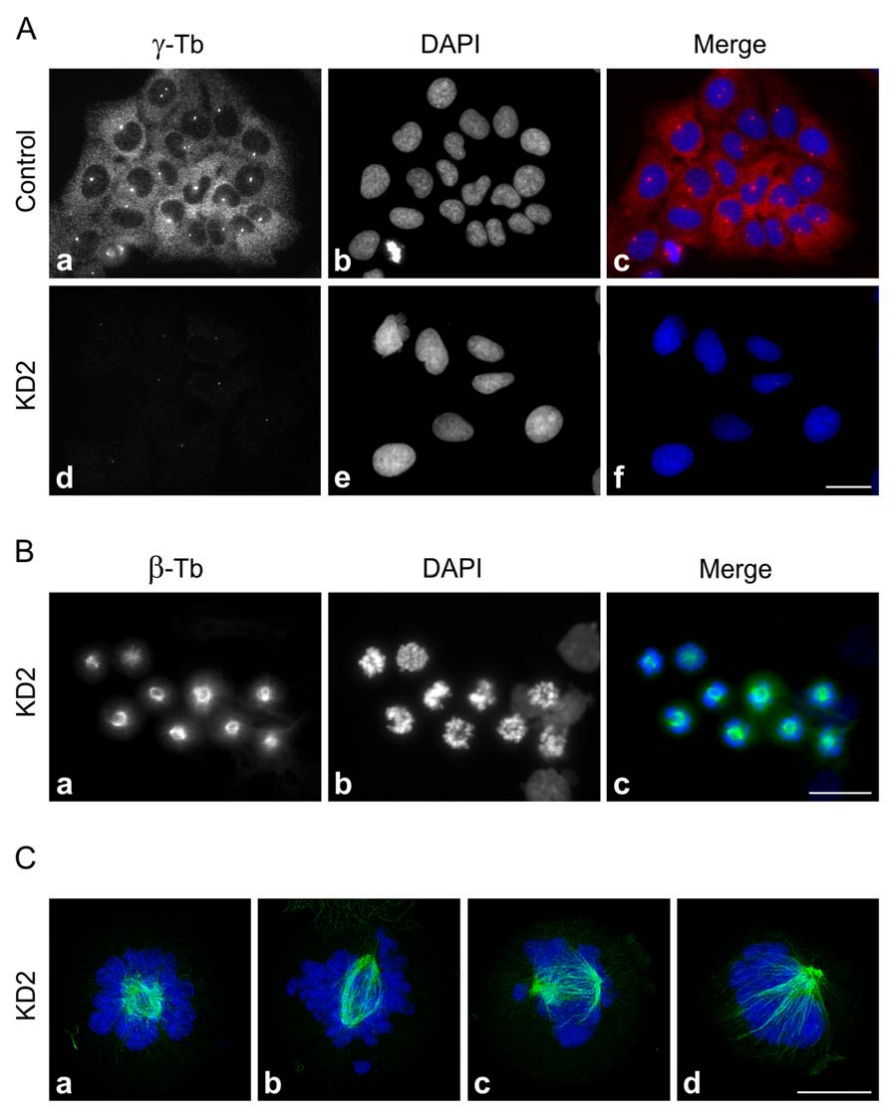
Depletion of human γ-tubulin 1 leads to mitotic spindle defects and metaphase arrest. (A) Interphase U2OS cells transfected with negative control siRNA (Control) or with γ-tubulin 1 specific siRNA (KD2). Cells were stained for γ-tubulin (a, d; red ). DNA was stained with DAPI (b, e; blue). Images of cells stained for γ-tubulin were captured under identical conditions and processed in exactly the same way. Scale bar 20 µm. (B) Aberrant spindle formation and metaphase arrest in U2OS cells depleted of γ-tubulin 1 (KD2). Cells were stained for β-tubulin (a; green). DNA was stained with DAPI (b; blue). Scale bar 20 µm. (C) Detailed images of aberrant mitotic spindles. Cells were stained for β-tubulin (a–d; green). DNA was stained with DAPI (a–d; blue).Maximum intensity projections of 30–40 deconvolved confocal z-sections spaced at 0.125 µm. Scale bar 10 µm.

To find out whether or not γ-tubulin 2 is able to take the place of γ-tubulin 1, we performed phenotypic rescue experiments in U2OS cells depleted of γ-tubulin 1 by RNAi. As demonstrated by immunoblotting, transfection of TUBG1-specific siRNAs (KD1 and KD2) led to a substantial reduction of total γ-tubulin content when compared to negative control cells (Fig. S3A). Noticeably, it means that γ-tubulin 1 is the dominant γ-tubulin in U2OS cells, because the specificity of both KD1 and KD2 siRNAs was verified *in silico* (NCBI BLAST) and by means of RT-qPCR (data not shown). Since KD2 siRNA proved to be more efficient, it was used in further experiments. Effective γ-tubulin depletion by KD2 siRNA was further confirmed by immunofluorescence microscopy (Fig. 2A). The most prominent phenotypic feature of γ-tubulin 1 depletion was mitotic arrest in metaphase (Fig. 2B), most likely induced by severe mitotic spindle defects (Fig 2C). Basically, cells in anaphase, telophase or cytokinesis were absent in the population of γ-tubulin 1-depleted cells.

**Figure 3:**
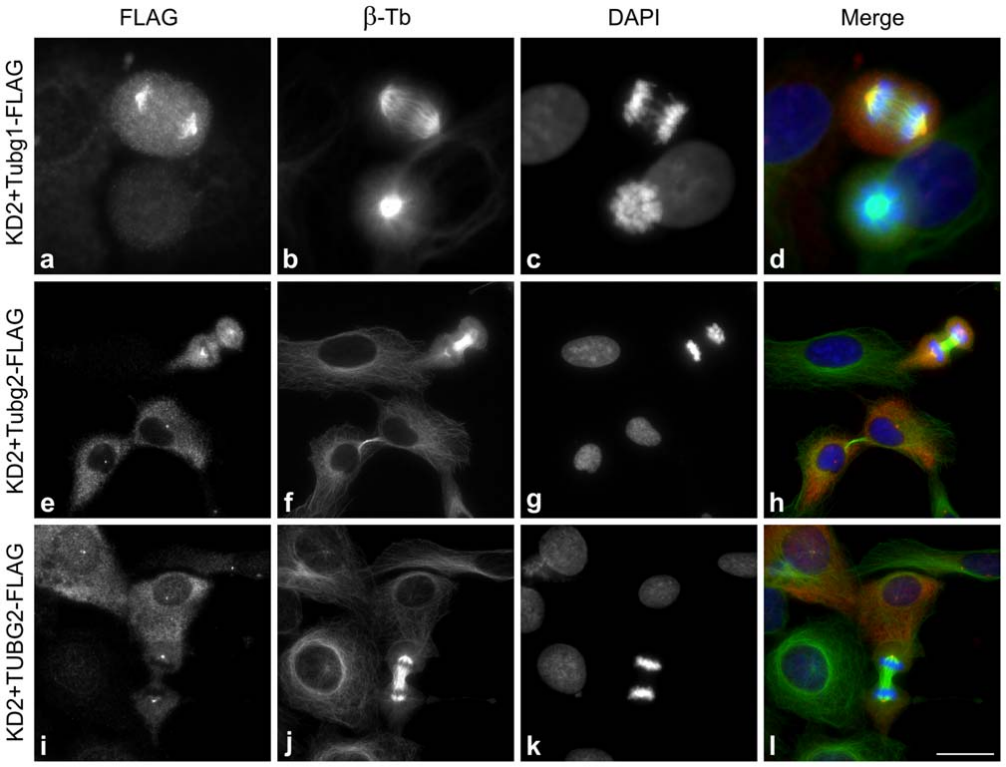
γ-Tubulin 2 restores normal mitotic spindle functioning in γ-tubulin 1-depleted cells. U2OS cells depleted of γ-tubulin 1 and expressing FLAG-tagged mouse γ-tubulin 1 (a-d, Tubg1-FLAG), mouse γ-tubulin 2 (e-h, Tubg2-FLAG) or human γ-tubulin 2 (i–l, TUBG2-FLAG) were stained for FLAG (a, e, i; red) and β-tubulin (b, f, j; green). DNA was stained with DAPI (c, g, k; blue). Scale bar 20 µm.

FLAG-tagged mouse γ-tubulin 1, used as a positive control, restored the original phenotype in γ-tubulin 1-depleted U2OS. Cells expressing exogenous γ-tubulin 1 were able to pass the spindle assembly checkpoint, as demonstrated by the presence of cells in anaphase, whereas the untrasfected cells were not (Fig. 3, a–d). Interestingly, FLAG-tagged mouse γ-tubulin 2 (Fig. 3, e–h) and FLAG-tagged human γ-tubulin 2 (Fig. 3, i–l) also rescued the normal mitotic division similarly to mouse γ-tubulin 1. Detailed microscopic examination of rescued cells revealed that they regained the ability to build properly arranged metaphase (Fig. S4, a–c) and anaphase (Fig. S4, d–f) mitotic spindles. Importantly, we failed to detect any mitotic spindle defects in the rescued cells. Immunoblot tests in performed rescue experiments confirmed an effective expression of FLAG-tagged γ-tubulins in γ-tubulin 1-depleted cells (Fig. S3B). These findings suggest that γ-tubulin 2 is capable of replacing γ-tubulin 1 during mitosis.

### γ-Tubulin 2 nucleates microtubules

**Figure 4:**
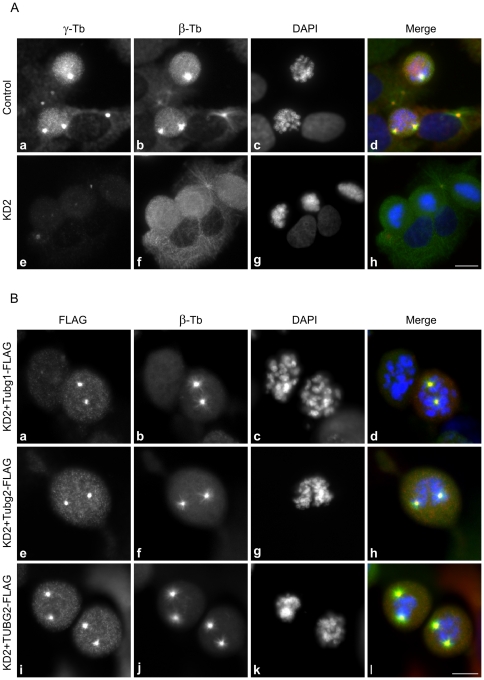
γ-Tubulin 2 rescues centrosomal microtubule nucleation in γ-tubulin 1-depleted mitotic cells. A) U2OS cells transfected with negative control siRNA (Control) or with γ-tubulin 1 specific siRNA (KD2) were treated with 10 µM nocodazole for 6 h and fixed after 3 min incubation in medium without nocodazole. Cells were stained for γ-tubulin (a, e; red ) and β-tubulin (b, f; green). DNA was stained with DAPI (c, g; blue). Fluorescence images of cells stained for γ-tubulin were captured under identical conditions and processed in exactly the same way. Scale bar 10 µm. (B) U2OS cells depleted of γ-tubulin 1 and expressing FLAG-tagged mouse γ-tubulin 1 (a–d, Tubg1-FLAG), mouse γ-tubulin 2 (e–h, Tubg2-FLAG) or human γ-tubulin 2 (i–l, TUBG2-FLAG) were treated with 10 µM nocodazole for 6 h and fixed after 3 min incubation in medium without nocodazole. Cells were stained for FLAG (a, e, i; red) and β-tubulin (b, f, j; green). DNA was stained with DAPI (c, g, k; blue). Scale bar 10 µm.

Taking advantage of the above described phenotypic rescue experimental set-up, we further investigated the microtubule nucleating capability of γ-tubulin 2 in microtubule regrowth experiments. The amount of γ-tubulin on prophase/metaphase centrosomes is significantly higher than that in interphase due to the process called centrosome maturation [25], [26]. We therefore first focused on mitotic centrosomes, where one could expect a prominent effect of γ-tubulin depletion on microtubule nucleation. Microtubules were depolymerized by nocodazole, washed by ice-cold PBS, and allowed to regrow before fixation and staining for β-tubulin. Mitotic cells became more abundant in the course of nocodazole treatment. While the regrowth of microtubules from centrosomes was easily observable in cells transfected with negative control siRNA (Fig. 4A, a–d), it was substantially delayed and/or impaired in γ-tubulin 1-depleted cells (Fig. 4A, e–h). Clearly recognizable microtubule asters were seen in 97% (n = 369) of negative control mitotic cells. In γ-tubulin 1-depleted cells, however, microtubule asters were indistinct and formed in only 18% (n = 274) of mitotic cells. As expected, FLAG-tagged mouse γ-tubulin 1 (positive control) rescued the microtubule aster formation in γ-tubulin 1-depleted cells (Fig. 4B, a–d). In accordance with our previous results, both FLAG-tagged mouse γ-tubulin 2 (Fig. 4B, e–h) and FLAG-tagged human γ-tubulin 2 (Fig. 4B, i–l) also rescued aster formation. Clear microtubule regrowth was observed in all γ-tubulin 1-depleted cells expressing exogenous γ-tubulin 2; it indicates that γ-tubulin 2 is capable of centrosomal microtubule nucleation in mitotic cells.

In order to strengthen the evidence of microtubule nucleation capability of γ-tubulin 2, we quantified microtubule formation *in vivo* by the tracking microtubule (+) ends marked by EB1-GFP in interphase U2OS cells (U2OS-EB1). For live cell imaging we used the shRNA system based on pLKO.1 vectors. Puromycin selection for 6 days made it possible to analyze only γ-tubulin-depleted cells. We constructed TUBG1-specific shRNA expressing vectors based on siRNAs (KD1 and KD2), and tested their effectivity by immunoblotting (Fig S5A). Since KD2 shRNA was found more efficient, further experiments were limited to that. Substantial γ-tubulin depletion by KD2 shRNA was confirmed by immunofluorescence microscopy (Fig. S5B). Additionally, we prepared TagRFP-tagged mouse γ-tubulin 1 (pmTubg1-TagRFP) and TagRFP-tagged human γ-tubulin 2 (phTUBG2-TagRFP) for phenotypic rescue experiments. TagRFP (pCI-TagRFP) served as control.

**Figure 5:**
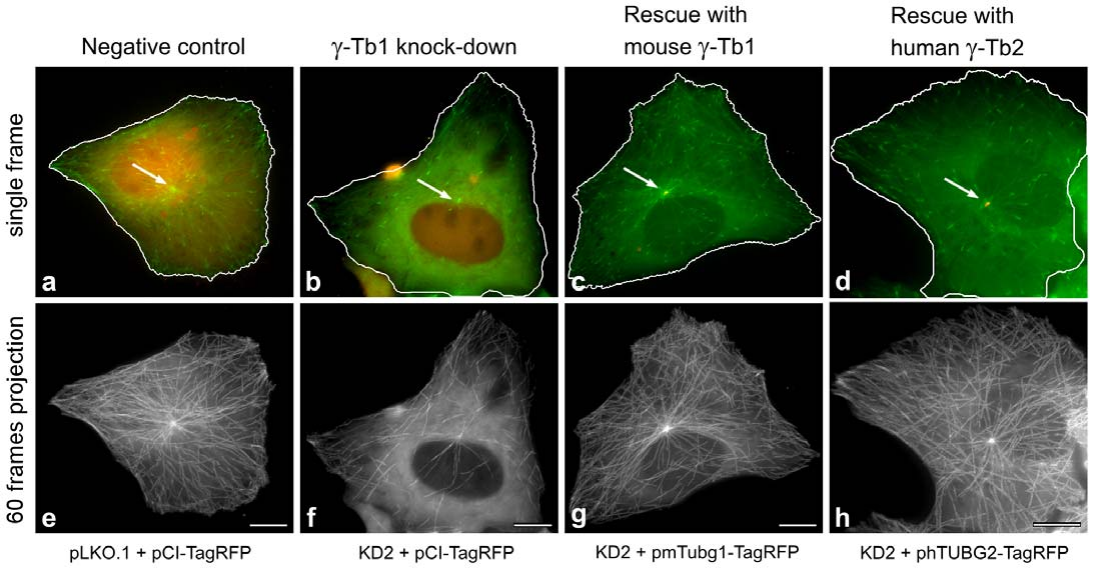
γ-Tubulin 2 rescues microtubule formation in γ-tubulin 1-depleted cells during interphase. Time-lapse imaging of U2OS-EB1 cells for quantitative evaluation of microtubule (+) end dynamics. Cells with depleted γ-tubulin 1 (KD2) expressing either TagRFP (pCI-TagRFP), mouse γ-tubulin 1 (pmTubg1-TagRFP) or human γ-tubulin 2 (phTUBG2-TagRFP). Cells with empty vector (pLKO.1) expressing TagRFP (pCI-TagRFP) served as negative control. (a–d) Still images of typical cells selected for evaluation. Only cells expressing both EB1-GFP (green) and TagRFP (red) or γ-tubulin-TagRFP fusions (red) were evaluated. In contrast to freely diffusible TagRFP (a, b), γ-tubulin-TagRFP fusions properly localized to MTOCs (c, d) marked by white arrows. (e–f) Maximum intensity projections of 60 consecutive time-frames from acquired time-lapse sequences. Note the markedly lower density of microtubule tracks in cell with depleted human γ-tubulin 1 (f). Microtubule track density is rescued in cells expressing exogenous mouse γ-tubulin 1 (g) or exogenous human γ-tubulin 2 (h). Scale bar 10 µm.

**Figure 6:**
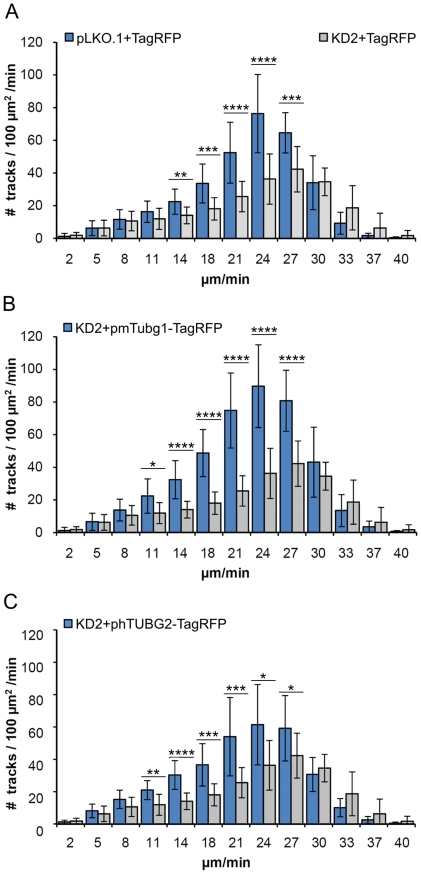
Quantitative evaluation of microtubule formation in phenotypic rescue experiments. Microtubule (+) end dynamics in U2OS-EB1 cells presented as velocity histograms. Cells with depleted γ-tubulin 1 (KD2) or negative control cells (pLKO.1), expressing either TagRFP (pCI-TagRFP), mouse γ-tubulin 1 (pmTubg1-TagRFP) or human γ-tubulin 2 (phTUBG2-TagRFP). (A) Comparison of negative control cells (pLKO.1+pCI-TagRFP; n = 19) with γ-tubulin 1 depleted cells (KD2+pCI-TagRFP; n = 15). (B) Comparison of cells rescued with mouse γ-tubulin 1 (positive control; KD2+pmTubg1-TagRFP; n = 18) with γ-tubulin 1 depleted cells. (C) Comparison of cells rescued with human γ-tubulin 2 (KD2+hTubg2-TagRFP; n = 19) with γ-tubulin 1 depleted cells. Data are from 3 independent experiments. Bars represent means±SD. Asterisks represent the p-values (p) of two-sided unpaired t-test (****, p<0.00001; ***, p<0.0001; **, p<0.001; *, p<0.01).

Following puromycin selection, transfected U2OS-EB1 cells were subjected to live cell imaging; time-lapse sequences of EB1-GFP dynamics were acquired only from cells coexpressing TagRFP or TagRFP-tagged proteins. Immunoblotting confirmed an effective expression of tagged γ-tubulins in γ-tubulin 1-depleted cells (Fig. S6). Results of typical experiments are presented in Fig. 5, where single-frame (Fig. 5, a–d) as well as 60-frame projections (Fig. 5, e–h) of time-lapse sequences are shown. While TagRFP was found in both cytoplasm and nuclei (Fig 5, a–b), TagRFP-tagged γ-tubulins were concentrated to MTOC (Fig. 5, c–d). This is more distinctly demonstrated in Fig. S7, where green and red channels are depicted separately. The density of microtubule (+) end tracks, reconstructed by maximum intensity projection of time-lapse sequences, was markedly reduced in γ-tubulin 1-depleted cells (Fig. 5, f) when compared with negative control cells (Fig. 5, e). This most likely reflects an impaired microtubule nucleation. In contrast, the density of EB1 tracks in cells rescued by exogenous mouse γ-tubulin 1 (Fig. 5, g) resembled that seen in negative controls cells (Fig. 5, e). Clear phenotypic rescue was also observed in cells expressing exogenous human γ-tubulin 2 (Fig. 5, h). These findings were confirmed by evaluation of statistical data as documented in histograms of the microtubule growth rates, where the number of EB1 tracks was normalized by the cell area and tracking time (Fig. 6). To compare whole populations of EB1 tracks in analyzed cells, we applied Bonferroni correction of p-values to velocity histograms (Fig. 6). Calculated p-values for differences among individual growth velocity groups were multiplied by the number of all growth velocity groups in the histogram (n = 13). Based on this correction, the number of EB1 tracks was significantly reduced in γ-tubulin 1-depleted cells when compared with negative control cells (p<0.0001, Fig. 6A). Conversely, the number of EB1 tracks was significantly higher in cells rescued by exogenous mouse γ-tubulin 1 (p<1.10^−6^, Fig. 6B) or human γ-tubulin 2 (p<1.10^−5^, Fig. 6C) than in γ-tubulin 1-depleted cells. Differences between negative control (blue columns in Fig. 6A) and γ-tubulin 2 expressing cells (blue columns in Fig. 6C) were statistically insignificant. Interestingly, the number of EB1 tracks in cells expressing exogenous mouse γ-tubulin 1 (blue columns in Fig. 6B) exceeded that seen in negative control (p<0.05; blue columns in Fig. 6A) or in cells expressing exogenous γ-tubulin 2 (p<0.05; blue columns in Fig. 6C). Taken collectively, our experimental data demonstrate that mammalian γ-tubulin 2 is able to nucleate microtubules and substitute for γ-tubulin 1 even in interphase cells.

### *Tubg2* is downregulated in mouse preimplantation development

**Figure 7:**
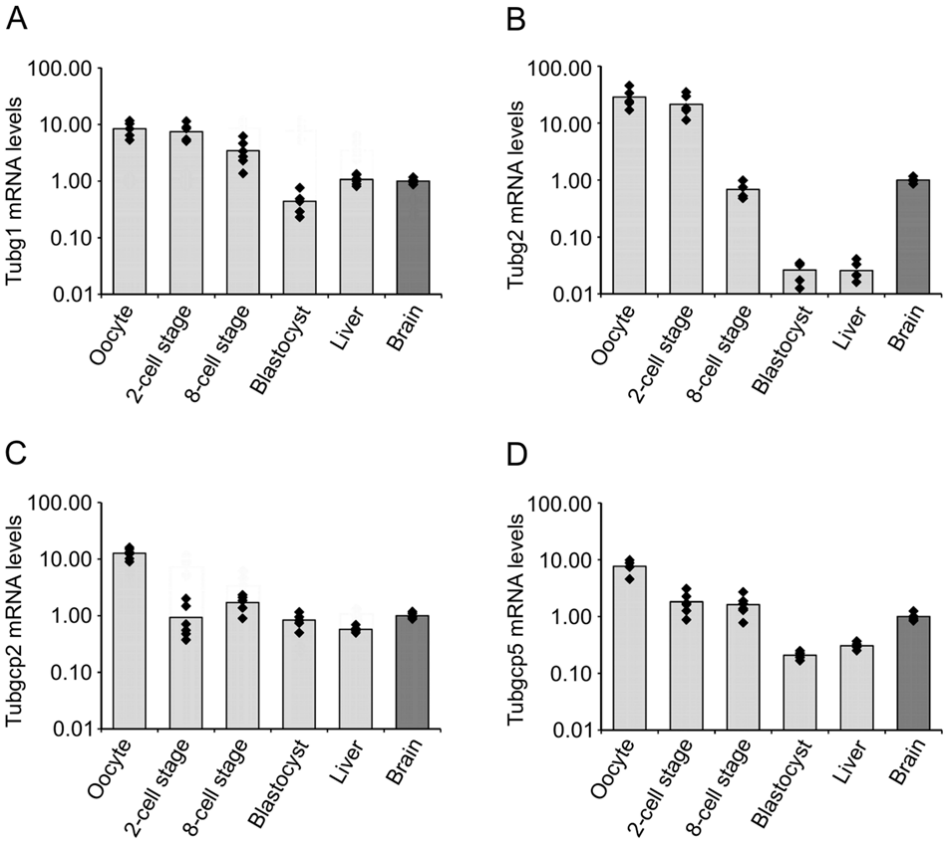
*Tubg2* mRNA level is decreasing during mouse preimplantation development. mRNA levels of *Tubg1* (A), *Tubg2* (B), *Tubgcp2* (C) and *Tubgcp5* (D) in mouse oocyte, 2-cell stage embryo, 8-cell stage embryo, blastocyst and liver relative to the level found in brain. Data are presented as mean fold change (columns) with individual samples displayed (diamonds). Three biological replicates were measured twice under identical conditions. Note that the Y-axis is in the logarithmic scale.

Since γ-tubulin 2 was capable to substitute for γ-tubulin 1 in cultured cells, its inability to do so in blastocysts [23] is intriguing. We therefore quantified by RT-qPCR the mRNA levels of *Tubg1* and *Tubg2* in mouse oocytes, 2-cell stage embryos, 8-cell stage embryos and blastocysts. Adult mouse liver and brain tissues served as controls, because *Tubg2* expression is high in brain and low in liver [23], [24]. Geometric mean of mouse peptidylprolyl isomerase A (*Ppia*) and mouse glyceraldehyde-3-phosphate dehydrogenase (*Gapdh*) mRNA levels were used for normalization. *Tubg1* mRNA level decreased 17 times, when 2-cell stage embryos were compared with blastocysts, and was almost equal in liver and brain (Fig 7A). In contrast, *Tubg2* mRNA level decreased dramatically by almost three orders of magnitude (815 times), when these two developmental stages were compared. *Tubg2* expression in blastocysts was comparable to that in liver and was 38 times lower than in brain (Fig. 7B). For comparison, mRNA levels were also ascertained for *Tubgcp2* and *Tubgcp5* that encode, respectively, GCP2 and GCP5 proteins. While *Tubgcp2* mRNA level remained relatively stable (Fig. 7C), that of *Tubgcp5* decreased 9 times when comparing the 2-cell stage embryos and blastocysts (Fig. 7D). Notably, the highest mRNA levels of tested genes were detected in oocytes, which probably reflects the high content of stored maternal mRNA [27]. Taken together, our data clearly show that *Tubg2* mRNA level is appreciably decreasing during mouse preimplantation development.

**Figure 8:**
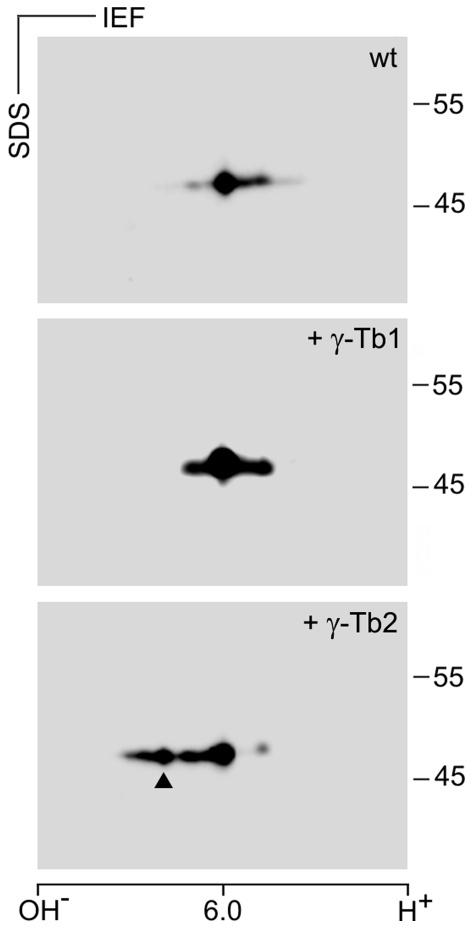
Electrophoretic distinction of mouse γ-tubulins on 2D-PAGE. Immunoblots of mouse P19 cell extracts separated by 2D-PAGE probed with antibody to γ-tubulin. Wild-type cells (wt), cells expressing exogenous untagged mouse γ-tubulin 1 (+γ-Tb1) or mouse γ-tubulin 2 (+γ-Tb2). Molecular mass markers (in kDa) are indicated on the right. The pI scale is shown along the bottom of the figure. IEF, isoelectric focusing. Arrowhead indicates the position of mouse γ-tubulin 2.

RT-qPCR analysis disclosed that blastocyst contains a very low amount of *Tubg2* mRNA. However, γ-tubulin 2 protein might still be present. To analyze the expression of *Tubg2* at the protein level, we first identified the positions of mouse γ-tubulin 1 and γ-tubulin 2 in samples separated by 2D-PAGE. Different antibodies reacting with both γ-tubulins were used for immunoblotting. The exact positions of γ-tubulin 1 and γ-tubulin 2 were determined by overexpression of, respectively, untagged mouse γ-tubulin 1 and γ-tubulin 2 in P19 cells, where *Tubg2* was undetectable by RT-qPCR (Fig. S8). Immunoblotting of untransfected and transfected cells with anti-γ-tubulin antibodies revealed that the signal of main γ-tubulin isoforms in P19 cells (Fig. 8, wt) was enhanced in cells overexpressing the γ-tubulin 1 (Fig. 8, +γ-Tb1). In cells overexpressing γ-tubulin 2, a new signal appeared in a more basic position compared to γ-tubulin1 isoforms (Fig. 8, +γ-Tb2). This was in agreement with theoretical isoelectric points for γ-tubulin 1 (5.66) and γ-tubulin 2 (5.80). These experiments demonstrate that mouse γ-tubulins can be easily discriminated on 2D-PAGE.

**Figure 9:**
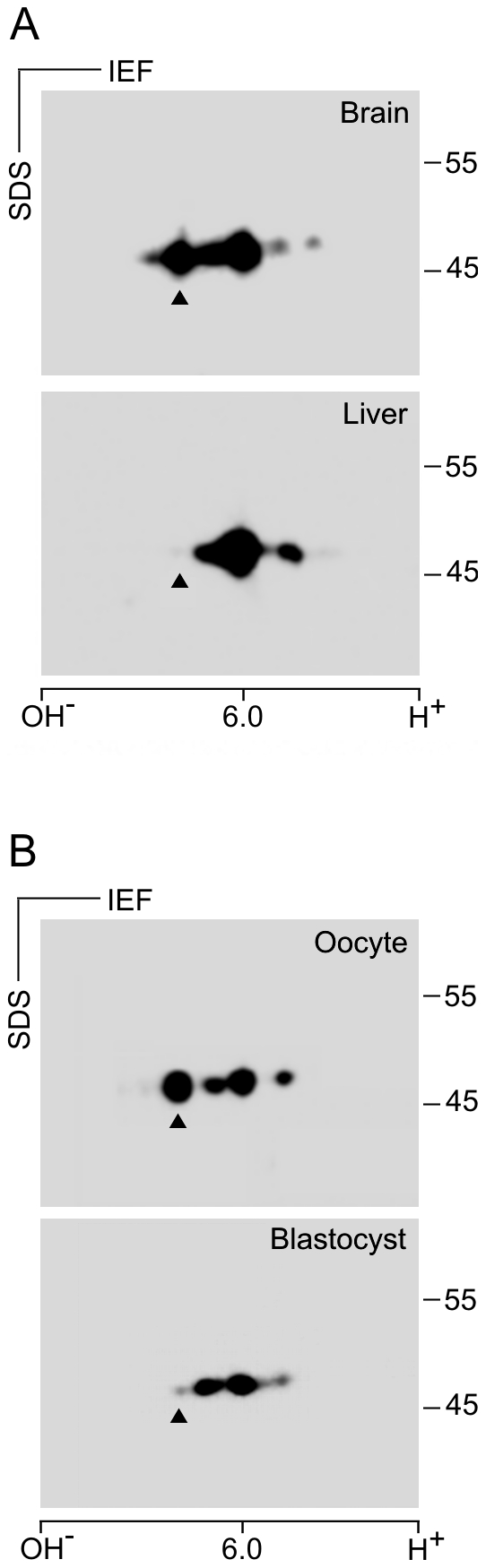
Differences in the expression of mouse γ-tubulin 2 protein in oocytes and blastocysts. Immunoblot analysis of tissue and cell extracts separated by 2D-PAGE with antibody to γ-tubulin. (A) Comparison of control adult mouse brain and liver. (B) Comparison of mouse oocytes and blastocysts. Molecular mass markers (in kDa) are indicated on the right. The pI scale is shown along the bottom of the figure. IEF, isoelectric focusing. Arrowheads indicate the position of γ-tubulin 2 as defined in Fig. 8.

To rule out the possibility that the isoelectric point of exogenous γ-tubulin 2 expressed in P19 cells substantially differs from that in mouse tissues, we compared the expression of γ-tubulins in mouse brain and mouse liver, where *Tubg2* expression is high and low, respectively [23], [24]. For this, we used immunoblotting after 2D-PAGE separation of samples containing similar total protein amounts. γ-Tubulin 1 was clearly detectable in both brain and liver. In contrast, a strong signal in the position of γ-tubulin 2 was detected merely in brain, whereas it was undetectable in liver (Fig. 9A). Again, these results correlated with data obtained in RT-qPCR experiments (Fig. 7B). The performed experiments confirmed that γ-tubulin 2 can be discriminated by 2D-PAGE also in mouse tissues. Using the same approach, we compared the expression of γ-tubulin 1 and γ-tubulin 2 in mouse oocytes and blastocysts. Samples were prepared from 150 fully grown oocytes at the GV stage and from 197 early blastocysts to ensure that the total protein amount in blastocyst sample was not underestimated. A fully grown oocyte (from adult animals) at GV stage contains approximately 30 ng of protein. Zona pellucida contributes to this amount some 4–5 ng [28]. An early blastocyst contains approximately 25 ng of protein [29]. γ-Tubulin 1 was clearly detectable in both oocytes and blastocysts. On the other hand, while there was a strong signal detectable in the position of γ-tubulin 2 in oocytes, the relevant signal in this position was dramatically reduced in blastocysts (Fig. 9B). Expression of γ-tubulin 2 at the protein level in oocytes and blastocysts thus correlated with its mRNA level (Fig. 7B). Collectively taken, these data strongly indicate that a very low amount of γ-tubulin 2 is present in wild-type blastocysts due to its transcriptional downregulation.

## Discussion

Mammalian γ-tubulins are encoded by two closely related genes [22], [24], and specific functions have been attributed to them. [23]. The molecular basis of suggested functional differences between γ-tubulins is however unknown. In this study we document that mammalian γ-tubulin 2 is able to nucleate microtubules and substitute for γ-tubulin 1. In addition, we show that *Tubg1* and *Tubg2* are differentially transcribed during mouse early embryogenesis, with *Tubg2* transcription being progressively downregulated.

In general, γ-tubulins are highly conserved proteins in all eukaryotes. At the amino acid sequence level, human γ-tubulin 1 and γ-tubulin 2, respectively, show 98.9% and 97.6% identity with the corresponding mouse isoforms (Table S1) [23]. To study the subcellular localization and function of human and mouse γ-tubulin 2, we have chosen human osteosarcoma cells U2OS. Because of their flat shape, they are excellent for immunofluorescence analysis and are easily transfectable. Moreover, the the selection of U2OS made it possible to answer the question whether or not the mouse γ-tubulin 2 is capable of replacing human γ-tubulin 1. We have used exogenously expressed FLAG-tagged mouse and human γ-tubulins to evaluate the subcellular localization of γ-tubulin 2 proteins and their interactions with GCPs. It was reported previously that exogenous mouse γ-tubulin 2 located to interphase and mitotic centrosomes in mouse Eph4 epithelial cells [23]. Our data corroborate this finding by showing that both human and mouse γ-tubulin 2 are recruited to interphase and mitotic centrosomes in human U2OS cells. By immunoprecipitation experiments we found that γ-tubulin 2 interacted with GCP2, an integral component of γTuSCs. Reciprocal coimmunoprecipitations of γ-tubulin 2 and GCP4 (T. Sulimenko, unpublished data) indicated that γ-tubulin 2 normally also incorporated in γTuRCs. We found no differences between γ-tubulin 1 and γ-tubulin 2 with regard to their localization and interactions. Intriguingly, antibody to GCP2 coimmunoprecipitated more endogenous than exogenous γ-tubulins (Fig. 1B, Fig. S2). A similar result was obtained with antibody to GCP4 (T. Sulimenko, unpublished data). This fact might indicate a slow turnover of γTuCs, because precipitations were performed 48 hours after transfection. Alternatively, FLAG tags might interfere with interaction of γ-tubulin with GCPs. However, this seems unlikely as FLAG tags were fused to the C-termini of γ-tubulins, which probably is not involved in the interaction with GCP2 and GCP3 [30]. Moreover, FLAG-tagged γ-tubulins rescued normal mitotic progression in γ-tubulin 1-depleted cells (Fig. 3).

The most remarkable phenotypic sign of γ-tubulin 1-depleted U2OS cells was arrest in metaphase caused by mitotic spindle defects such as monopolar or collapsed spindles (Fig. 2C), previously described in mammalian cells depleted of γ-tubulin [11], [23], [31]. Similar defects were detected in cells in which γ-tubulin localization to centrosomes, mitotic spindle and mitotic chromatin was damaged by depletion of γTuRC recruitment factors like GCP-WD/NEDD1 [11], [31] or components of augmin complex [32], [33]. As expected, the observed phenotype was reverted by expression of mouse γ-tubulin 1. Both human and mouse γ-tubulin 2 likewise rescued the normal mitotic progression in γ-tubulin 1-depleted cells, indicating that mammalian γ-tubulin 2 is able to substitute for γ-tubulin 1 *in vivo* (Fig. 3). Consistent with these findings are the results of microtubule regrowth experiments on mitotic cells which reveal that γ-tubulin 2 does have microtubule nucleating capability (Fig. 4). We used only KD2 siRNA and corresponding shRNA for phenotypic rescue experiments, because it was more efficient than KD1 (Fig. S3A, Fig. S5A) and its specificity was verified in an independent study [34]. Rescue experiments also ruled out potential off-target RNAi effects.

When testing the microtubule (+) end dynamics in γ-tubulin 1-depleted cells, we observed a significant reduction in the number of EB1 tracks in interphase cells (Fig. 5, f; Fig. 6A), a sign of impaired microtubule nucleation. Alternatively, reduction in the EB1 track number might be explained by changes in microtubule dynamics; the nucleation is not affected but the fraction of growing microtubules relative to pausing or depolymerizing microtubules is diminished. Although one cannot exclude a potential contribution of impaired microtubule (+) ends dynamics to the observed phenotype, we consider this possibility much less probable because it has been previously demonstrated by regrowth experiments that microtubule nucleation is impaired and/or delayed in interphase cells depleted of γ-tubulin [31]. We therefore conclude that γ-tubulin 2 is able to nucleate microtubules also in interphase cells. Interestingly, a higher number of EB1 tracks was in γ-tubulin 1-depleted cells expressing exogenous γ-tubulin 1 than in cells expressing exogenous γ-tubulin 2 (Fig. 6). It might imply that for interphase cells γ-tubulin 1 is a more potent nucleator of microtubules than γ-tubulin 2. However, no corresponding differences in microtubule regrowth were observed in mitotic cells (Fig. 4), where centrosomes are highly enriched with γTuCs [25], [26], and where consequently the potential differences in nucleation capability ought to be stronger. In addition, statistical significance (p<0.05) of this difference is relatively low. We therefore do not think that γ-tubulin 1 and γ-tubulin 2 substantially differ in nucleation activity.

Functional redundancy of mammalian γ-tubulins was expected because of their high sequence similarity [22]. Importantly, only 6 amino acids specific for γ-tubulin 1- or γ-tubulin 2 are conserved in the majority of mammalian species. They are located in two clusters in helixes H11 (3 amino acids) and H12 (3 amino acids) of γ-tubulins (Table S2). These regions might be important for hypothetical divergent functions of mammalian γ-tubulins. However, when γ-tubulin in fission yeast was replaced by human γ-tubulin 1, with all three γ-tubulin 1-specific amino acids in helix H11 (R390, T391, R393) or one amino acid in helix H12 (I427) mutated to alanines, no deleterious effect on cell viability was observed [35]. It indicates that these regions are not essential for conserved γ-tubulin functions; this is in line with our data suggesting that γ-tubulin 2 is able to substitute for γ-tubulin 1.

Yuba-Kubo et al. reported that γ-tubulin 2 is expressed in the wild-type mouse blastocyst [23]. In contrast, our 2D-PAGE analysis indicates that there is very low level of γ-tubulin 2 protein in the wild-type blastocyst, whereas γ-tubulin 1 is abundant (Fig. 9). This is in a good agreement with our RT-qPCR data, indicating that *Tubg2* mRNA level is dramatically decreasing during preimplantation development unlike mRNA levels of *Tubg1*, *Tubgcp2* and *Tubgcp5* (Fig. 7). The reason for such discrepancy is unclear. Previously blastocysts were analyzed only by immunoblotting after one-dimensional PAGE. Anti-γ-tubulin antibody recognized two bands that were supposed to represent γ-tubulin 1 and γ-tubulin 2 [23]. However, reported separation of γ-tubulins in blastocysts by SDS-PAGE need not reflect only the presence of different genes, but proteolysis or posttranslational modification(s) as well.

A common fate of the members of duplicate-gene pairs is the partitioning of tissue-specific patterns of expression of the ancestral gene [36]. Analyses of expression of mammalian γ-tubulin genes showed differential expression in many tissues [23], [24]; the same holds also for this study. It suggests that an important mechanism acting on γ-tubulin gene pair is the subfunctionalization. It was reported that some gene segments of γ-tubulin genes had been evolving together in the process known as „concerted evolution“ [24]. It was proposed that concerted evolution might have been operative to maintain perfect homology at essential binding sites. Indeed, exons 2–3 and 7–10 of the two γ-tubulin genes homogenized by concerted evolution [24] encode regions which are probably critical for interaction of γ-tubulin with GCP2 and GCP3 [30]. Thus, concerted evolution together with subfunctionalization foster the preservation of highly similar and functionally redundant γ-tubulin genes in mammalian genomes [24].

Our data allow an alternative interpretation of *Tubg1^-/-^* and *Tubg2^-/-^* phenotypes previously described in mice [23]. Endogenous γ-tubulin 2 cannot rescue γ-tubulin 1 deficiency in Tubg1^-/-^ blastocyst, even though it can nucleate microtubules, because it is not present in a sufficient amount. It was previously reported that knock out of single gene resulted in overexpression of related genes [37]–[39]. Our data do not strictly exclude the possibility that γ-tubulin 2 expression could be up-regulated in *Tubg1^-/-^* blastocysts, however, γ-tubulin 2 may be insufficient to fully replacer the lacking γ-tubulin 1. On the other hand, whole-mount immunostaining with anti-γ-tubulin antibody in Tubg1^-/-^ blastocyst cells did not identify any γ-tubulin 2-positive foci, even though one pericentrin-positive focus occurred in each cell [23]. It was suggested that γ-tubulin 1 was necessary for recruitment of γ-tubulin 2 to blastocyst centrosomes [23]. We propose that such observation can be alternatively explained by the absence of γ-tubulin 2 at the blastocyst stage both both in wild type and Tubg1^-/-^ embryos. Behavioral abnormalities of Tubg2 ^-/-^ mice do not necessarily imply unknown function(s) of γ-tubulin 2. They might also reflect a reduction of total γ-tubulin in brain of *Tubg2^-/-^* mice, since *Tubg2* is highly expressed in the brain [23], [24] as demonstrated also in this study. Yet, we cannot exclude the possibility that brain γ-tubulin 2 has some additional still unknown function(s). Thorough phenotype analysis of *Tubg2^-/-^* mice could shed more light on γ-tubulin 2 function(s) in brain and its development. Further, elucidation of transcriptional regulation of γ-tubulin genes would by very important not only from the developmental point of view but also with respect to tumorigenesis. Significantly higher expression of γ-tubulin was found in high- versus low-grade gliomas, common brain cancers [40], [41].

In conclusion, the findings indicate that mammalian γ-tubulin 2 is able to nucleate microtubules and substitute for γ-tubulin 1. Although γ-tubulins are differentially expressed during mouse early embryogenesis and in adult tissues, they are functionally redundant with respect to their nucleation activity.

## Materials and Methods

### Ethics statement

All mice were maintained in accordance with the Institute of Molecular Genetics Guidelines. Experiments were approved by the Committee on the Ethics of Animal Experiments of the Institute of Molecular Genetics (permit number 18/2009).

### Cell cultures and transfections

Human osteogenic sarcoma cells U2OS, human glioblastoma cell line T98G, mouse embryonal carcinoma cells P19, mouse neuroblastoma Neuro-2a and mouse embryonal fibroblasts NIH 3T3 were obtained from the American Type Culture Collection. Human kidney embryonal cells HEK293-FT (HEK) were from Promega Biotec. Mouse bone marrow-derived mast cell line (BMMC) was kindly provided by M. Hibbs (Ludwig Institute for Cancer Research, Melbourne, Australia). Cells were cultured in Dulbecco's modified Eagle's medium (DMEM) containing 10% fetal bovine serum, penicillin (100 units/ml), and streptomycin (0.1 mg/ml). Cells were grown at 37°C in 5% CO_2_ in air, and passaged every 2 or 3 days using 0.25% trypsin/0.01% EDTA in PBS. BMMC were cultured in RPMI 1640 medium supplemented with serum, antibiotics and interleukin 3 (PeproTech) as described previously [42].

U2OS cells were transfected with 2.5 µg (single plasmid) or 4 µg (cotransfection of 2 plasmids) DNA/well in a 6-well plate using Lipofectamine LTX reagent (Invitrogen) (DNA[µg]: LTX [µl] ratio was 1∶2.5) and Opti MEM medium (Gibco) according to the manufacturer's instruction. After 12 h, the transfection mixture was replaced with fresh complete medium, and cells were incubated for 48 h. HEK cells were transfected with 17 µg DNA per 9-cm tissue culture dish using 51 µg polyethylenimine (Polysciences) and serum-free DMEM. After 24 h, the transfection mixture was replaced with fresh medium supplemented with serum, and cells were incubated for additional 24 h.

Some of the U2OS cells, growing on coverslips, were treated with 10 µM nocodazole (Sigma) for 6 h. Afterwards, cells were washed 5 times in ice-cold PBS, transferred to new medium and incubated for 3 min at 28°C before fixation.

### Mouse oocytes, embryos and tissues

Oocytes and embryos were obtained from 6–8 week old C57BL/6 mice. Fully grown germinal vesicle (GV) oocytes were liberated from ovaries by puncturing the antral follicles with syringe needle and collected in M2 medium (Sigma) containing 0.2 mM isobutylmethylxanthine (IBMX; Sigma). To obtain preimplantation embryos, mice were superovulated with 5 IU of Folligon (pregnant mare serum gonadotropin; PMSG) (Intervet) followed by stimulation with 5 IU of human chorionic gonadotrophin (hCG, Sigma) 47 hours post-PMSG. The stimulated mice were mated with 8–10 week old C57BL/6 males immediately after hCG injection. Two-cell and eight-cell stage embryos were collected 48 and 68 hours post-hCG, respectively, by flushing the oviducts with M2 medium. Blastocyst stage embryos were collected 96 hours post-hCG by flushing the uteri with M2 medium. Oocytes and embryos were washed 5 times in PBS prior to transfer into TRI reagent (Ambion) for RNA isolation or into buffer for 2D-PAGE. Liver and brain were dissected from 6–8 week old female C57BL/6 mice.

### DNA constructs

Total RNA from BALB/c adult mouse brain or from human cell line T98G was isolated by the RNeasy Mini kit (QIAGEN) according to the manufacturer's directions. The purity and integrity of the RNA preparations were checked using Experion automated electrophoresis system for microfluidic chip-based analysis and RNA StdSens analysis kit (Bio-Rad Laboratories). The quantity of RNA was checked by Nanodrop spectrophotometer (NanoDrop Technologies). Reverse transcription was performed with oligo(dT) primers and SuperScript III Reverse Transcriptase kit (Invitrogen).

The full length human γ-tubulin 2 (*TUBG2*, Refseq ID: NM_016437) was amplified by PCR using forward 5′- GCCCACGTCTGAAGAGCGATGC-3′ and reverse 5′-CTGGAGATGAACCAAGAAGGGTTG-3′ primers and T98G cell cDNA as template. The full length mouse γ-tubulin 1 (*Tubg1*, Refseq ID: NM_134024) and mouse γ-tubulin 2 (*Tubg2*, Refseq ID: NM_134028) were amplified by PCR using the following specific primers - *Tubg1*: forward 5′-GAGAGACTGCAACGCCGATGTCTG-3′ and reverse 5′-TTGTGAGGTCCCTGATCTGTGCTC-3′; *Tubg2*, forward 5′- GGCAGGAGTTCCTCTCAGTCGTGAC-3′ and reverse 5′-TGTGAGGCGAAGTTGGGTCAGAG-3′ - and mouse brain cDNA as template.

PCR products were ligated into pCR 3.1 vector (Invitrogen) by TA-cloning method. Sequencing revealed that the M413V variant of human γ-tubulin 2 was isolated (refSNP ID: rs1046097). The constructed vectors (pCR-hTUBG2, pCR-mTubg1, pCR-mTubg2) served as templates for additional PCRs with following specific forward and reverse primers carrying *Eco*RI/*Sal*I restriction sites (underlined): human *TUBG2*, forward 5′-CTGAATTCCACGTCTGAAGAGCGATGC-3′ and reverse 5′-TCAAGTCGACCTGCTCCTGGGTGCC-3′; mouse *Tubg1*: forward 5′-ACGAATTCTGCCTGAGGAGCGATGC-3′ and reverse 5′-TCAAGTCGACCTGCTCCTGGGTGCC-3′; mouse *Tubg2*: forward 5′- CTGAATTCGGTCTGATCGGCGATGC-3′ and reverse 5′-TCAAGTCGACCTGCTCCTGGGTGCC-3′. PCR products without stop codon were digested with *Eco*RI/*Sal*I restriction enzymes and inserted into pFLAG-CMV5a vector (Sigma) resulting in C-terminally FLAG-tagged human γ-tubulin 2 (phTUBG2-FLAG), mouse γ-tubulin 1 (pmTubg1-FLAG) and mouse γ-tubulin 2 (pmTubg2-FLAG). Constructs encoding C-terminally FLAG-tagged human γ-tubulin 1 (phTUBG1-FLAG) or mouse Fyn (pFyn-FLAG) were described previously [9].

Complete coding sequences of mouse *Tubg1* and *Tubg2* were cut out from pCR-mTubg1 and pCR-mTubg2, respectively, by *Eco*RI and inserted into pCI-NEO (Promega) to create vectors expressing untagged mouse γ-tubulin 1 (pCI-mTubg1) and γ-tubulin 2 (pCI-mTubg2).

Coding sequence of monomeric red fluorescent protein TagRFP-T (GenBank: EU582019.1) was amplified from pcDNA3.1-TagRFP (kind gift of Dr. R.Y. Tsien, HHMI at the University of California, San Diego, USA) by PCR with the following specific forward and reverse primers carrying *Sal*I and *Not*I restriction sites (underlined): forward 5′-AGTCGAC *GGAGGTGGTGGAGGT*ATGGTGTCTAAGGGCGAAGA-3′ (added 5x glycine coding motif in *italic*) and reverse 5′- TGCGGCCGCTTACTTGTACAGCTCGTCCATGCCA -3′. PCR products were ligated into pCR 2.1 vector (Invitrogen) by TA-cloning method. TagRFP coding sequence was digested from this vector using *Sal*I/*Not*I restriction enzymes and inserted into pCI-Neo (Promega) resulting in a vector encoding TagRFP (pCI-TagRFP) and allowing construction of C-terminally TagRFP-tagged fusion proteins. Coding sequences of mouse *Tubg1* and human *TUBG2* without stop codon were cut out from pmTubg1-FLAG and phTUBG2-FLAG, respectively, by *Eco*RI/*Sal*I restriction enzymes and ligated into pCI-TagRFP resulting in vectors encoding TagRFP-tagged mouse γ-tubulin 1 (pmTubg1-TagRFP) and human γ-tubulin 2 (phTUBG2-TagRFP). All constructs were verified by sequencing.

U2OS cells stably expressing EB1-GFP (U2OS-EB1) were obtained by transfection of cells with pEB1-GFP, obtained from Dr. Y. Mimori-Kiyosue [43], and selection in 1.1 mg/ml geneticin (G418, Sigma) for 2 weeks. Cells were then diluted to one cell/well on 96-well plate and allowed to grow for 2 weeks. Homogeneous colonies expressing EB1-GFP were propagated.

### Antibodies

The following anti-peptide antibodies prepared to human γ-tubulin were used: mouse monoclonal antibodies TU-30 (IgG1) and TU-32 (IgG1) to the sequence 434–449 [44]; monoclonal antibody GTU 88 (IgG1; Sigma, T6657) and rabbit antibody (Sigma, T5192) to the sequence 38–53. The anti-γ-tubulin antibodies react with both γ-tubulin 1 and γ-tubulin 2 in mouse and human. β-Tubulin was detected with monoclonal antibody TUB 2.1 conjugated with FITC (IgG1; Sigma F2043) and pericentrin with rabbit antibody (Covance PRB-432C). Rabbit antibodies to GAPDH (G9545) and FLAG peptide (F7425) as well as monoclonal antibody M2 (IgG1) to FLAG peptide (F1804) were from Sigma. Monoclonal antibodies to GCP2 protein, GCP2-01 (IgG2b) and GCP2-02 (IgG1), were described previously [9]. Monoclonal antibody to GCP4 (IgG1) was from Santa Cruz (sc-271876). Monoclonal antibody NF-09 (IgG2b) to neurofilament NF-M protein [45] and rabbit antibody to non-muscle myosin BT-561 (Biomed Techn. Inc) served as controls.

The Cy3-conjugated anti-mouse and anti-rabbit antibodies were from Jackson Immunoresearch Laboratories. Anti-rabbit antibody conjugated with Alexa 488 was from Invitrogen. Secondary horseradish peroxidase-conjugated antibodies were from Promega.

### RNAi

U2OS cells in 6-well plates were transfected with siRNAs (final concentration 20 nM) using Lipofectamine RNAi MAX (Invitrogen) according to the manufacturer's instruction. Five siRNAs (Ambion/Applied Biosystems) that target the regions present in human γ-tubulin 1, namely, (5′-GGGAGAAAAGATCCATGAG-3′; siRNA ID #9227), (5′-CGCATCTCTTTCTCATATA-3′; siRNA ID #120194), (5′-GGACATTTTTGACATCATA -3′; siRNA ID #9317), (5′-GAACCTGTCGCCAGTATGA-3′; siRNA ID #120784), (5′-GGTATCCTAAGAAGCTGGT-3′; siRNA ID #9396) were tested. Maximal depletion was reached by transfecting the siRNA twice with a 72-h time interval and harvesting cells 72 h after the second transfection. Negative control siRNA was from Ambion/Applied Biosystems (Silencer Negative Control #1 siRNA).

Immunoblotting and immunofluorescence analyses revealed that the highest reduction of γ-tubulin was obtained with siRNA ID#9396 (KD1) and siRNA ID#120194 (KD2; results not shown). These siRNAs were used for some phenotypic rescue experiments. In that case siRNA was mixed with the plasmid of interest and transfected into cells which already underwent the first round of 72 h-long siRNA treatment, using Lipofectamine LTX. Cells were analyzed 72 h after the second transfection.

The selected siRNAs were also used for construction of shRNA vectors based on pLKO.1 (Addgene, #8453) that enabled puromycin selection. Corresponding sense and antisense oligonucleotides were synthetized by Sigma-Aldrich: 9396sh-sense 5′-CCGGGGTATCCTAAGAAGCTGGTTTCTCGAGACCAGCTTCTTAGGATACCTGTTTTTG-3′, 9396-antisense 5′-AATTCAAAAACAGGTATCCTAAGAAGCTGGTCTCGAGAAACCAGCTTCTTAGGATACC-3′; 120194sh-sense 5′- CCGGCGCATCTCTTTCTCATATATTCTCGAGTATATGAGAAAGAGATGCGTGTTTTTG-3′, 120194sh-antisense 5′- AATTCAAAAACACGCATCTCTTTCTCATATACTCGAGAATATATGAGAAAGAGATGCG-3′. Sense and antisense oligonucleotides, at final concentration 45 µM of each, were annealed in 1x NEB 2 buffer (New England Biolabs) by initial warming up to 95°C followed by slow (3 h) cooling down to the room temperature. Annealed oligonucleotides were inserted into pLKO.1 previously linearized with *Age*I/*Eco*RI resulting in human TUBG1 shRNA expressing vectors p9396sh (KD1) and p120194sh (KD2). Correct sequences of all vectors were verified by sequencing.

shRNA vectors were transfected in the same way as other plasmids used in the study. To select shRNA expressing cells, the transfection mixture was replaced with fresh complete medium 12 h later. Puromycin (Sigma) at final concentration 2.5 µg/ml was added after 12 h incubation, and cells were selected in puromycin for 6 days before analysis.

### Reverse transcription quantitative real-time PCR (RT-qPCR) analysis

Total cellular RNA was extracted in three independent isolations from 20 mouse oocytes and 5–10 embryos using TRI reagent (Ambion) according to manufacturer's instructions. Three independent isolations of total cellular RNA were also made from four mouse cell lines Neuro2a, P19, BMMC and 3T3 using RNeasy Mini kit (QIAGEN). In three independent experiments mouse livers and brains were frozen in liquid nitrogen, homogenized under liquid nitrogen using mortar and pestle, and total RNA was extracted from 10–15 mg of homogenized tissue using RNeasy Minikit (QIAGEN). Concentration of the purified RNA was determined with spectrophotometer NanoDrop (Thermo Scientific). The quality of RNA was checked on Agilent 2100 Bioanalyzer. All RNA samples were of good qPCR quality (RNA integrity number [RIN] ≥7.6 for all tissue samples; RIN≥9.8 for all cell lines samples). Purified RNA was stored at −70°C. Purified RNAs from oocytes and embryos were converted to cDNA immediately after isolation. RNA from each sample was converted to cDNA using the ImProm-II RT kit (Promega). For tissues and cell lines, each reaction sample (20 µl) contained 1 µg RNA, random hexamer primers (25 ng/ µl), ImPROM-II reaction buffer, 5.6 mM MgCl_2_, dNTP mix (0.5 mM each dNTP), 0.5 µl RNasin and 1 µl ImProm-II reverse transcriptase. For oocytes and embryos, all isolated RNA was used for reverse transcription.

Quantitative PCR was performed with gene-specific primers for mouse γ-tubulin 1 (*Tubg1*, NM_134024), mouse γ-tubulin 2 (*Tubg2*, NM_134028), mouse GCP2 (*Tubgcp2*, NM_133755), mouse GCP5 (*Tubgcp5*, NM_146190), mouse peptidylprolyl isomerase A (*Ppia*, NM_008907) and mouse glyceraldehyde-3-phosphate dehydrogenase (*Gapdh*, NM_008084). All primers were tested *in silico* by NCBI BLAST to amplify specific targets. Primer sequences are summarized in Table S3. Oligonucleotides were from East Port (Prague, Czech Republic).

Quantitative PCRs were carried out on LightCycler 480 System (Roche). Each reaction (5 µl) consisted of 2.5 µl LightCycler® 480 SYBR Green I Master (Roche), 0.5 µl mixed gene-specific forward and reverse primers (5 µM each) and 2 µl diluted cDNA. cDNA samples from brain, liver and cell lines were diluted 1∶50. In case of oocyte and embryonal cDNA samples, the used amounts of cDNA per reaction corresponded to 1/4 of oocyte, 1/2 of 2-cell stage embryo, 1/10 of 8-cell stage embryo and 1/10 of blastocyst. Calibration curves for tested genes were made by serial dilutions (dilution factor 4) of brain cDNA. Each sample was run in duplicate. Thermocycling parametres are described in Text S1. Identity of PCR products was verified by sequencing.

### Preparation of cell extracts

Whole-cell extracts for SDS-PAGE were prepared by rinsing the cells twice in Hepes buffer (50 mM Hepes adjusted to pH 7.6 with NaOH, 75 mM NaCl, 1 mM MgCl_2_ and 1 mM EGTA), scraping them into Hepes buffer supplemented with protease (Roche; Complete EDTA-free protease mixture) and phosphatase (1 mM Na_3_VO_4_ and 1 mM NaF) inhibitors, and solubilizing in hot SDS-sample buffer [46] without bromphenol blue and boiling for 5 min.

When preparing the extracts for immunoprecipitation, cells were rinsed twice in cold Hepes buffer and extracted for 10 min at 4°C with Hepes buffer supplemented with protease and phosphatase inhibitors and 1% Nonidet P-40. The suspension was then spun down (20,000 *g*, 15 min, 4°C).

For preparation of samples for 2D-PAGE, oocytes and blastocysts were directly lysed in 2D-PAGE sample buffer [47]. Brain and liver were mixed with cold Hepes buffer supplemented with protease and phosphatase inhibitors in tissue/buffer ratio 1∶10 and homogenized in teflon/glass grinder. The suspension was then spun down (20,000 *g*, 15 min, 4°C) and 10–15 µl aliquots of supernatant were mixed with 200 µl of sample buffer. Similarly, 20 µl aliquots of supernatants from 1% NP-40 extracts of P19 cells were mixed with 200 µl of sample buffer.

Protein quantifications in lysates and SDS-PAGE-samples were performed, respectively, with bicinchoninic acid assay and silver dot assay [48].

### Immunoprecipitation, gel electrophoresis and immunoblotting

Immunoprecipitation from 1% NP-40 extracts was performed as described [49]. Cell extracts were incubated with beads of protein A (Pierce, Rockford, IL) saturated with: (I) rabbit antibody to FLAG, (II) monoclonal antibody GCP2-01 (IgG2b) to GCP2 (III) rabbit antibody to non-muscle myosin (negative control), (IV) monoclonal antibody NF-09 (IgG2a; negative control) or with (V) immobilized protein A alone. Gel electrophoresis and immunoblotting were performed using standard protocols. Two-dimensional electrophoresis (2D-PAGE) was performed as described [47] using for the first dimension 7 cm long Immobiline DryStrip gels with a linear pH 4-7 gradient (Amersham Biosciences). Comparable protein amounts were loaded in case of P19, liver and brain extracts (∼25 µg).

For immunoblotting, rabbit antibodies to GAPDH, γ-tubulin (Sigma T5192) and FLAG peptide (Sigma F7425) were diluted 1∶20,000, 1∶5,000 and 1∶2,000, respectively. Monoclonal antibodies to γ-tubulin (GTU88) and GCP4 were diluted 1∶10,000 and 1∶2,000, respectively. Monoclonal antibodies to γ-tubulin (TU-32) and GCP2 (GCP2-02), in the form of spent culture supernatants, were diluted 1∶10. Peroxidase-conjugated secondary antibodies were diluted 1∶10,000. Bound antibodies were detected by SuperSignal WestPico Chemiluminescent reagents (Pierce).

### Immunofluorescence

Immunofluorescence staining was performed as previously described [50]. Samples were fixed in methanol at −20°C, air-dried and washed in PBS. Rabbit antibodies to FLAG peptide and pericentrin were diluted 1∶1000 and 1∶750, respectively. Monoclonal antibodies to FLAG peptide and β-tubulin were diluted 1∶1,000 and 1∶100, respectively. Monoclonal antibody TU-30 to γ-tubulin was used as spent culture medium diluted 1∶50. Cy3-conjugated anti-mouse and ant-rabbit antibodies were diluted 1∶1,000, Alexa 488-conjugated anti-rabbit antibody was diluted 1∶200. For double-label immunofluorescence, coverslips were incubated separately with the primary antibodies, and simultaneously with the secondary conjugated antibodies. The preparations were mounted in MOWIOL 4–88 (Calbiochem) supplemented with 4,6-diamidino-2-phenylindole (DAPI, Sigma) to label nuclei, and examined on Delta Vision Core system (Applied Precision) equipped with 60x/1.42 NA oil-immersion objective. Optical z-sections were acquired in 0.25–0.30 µm steps. Z-stacks were deconvolved by a built-in deconvolution program (Softworx) using default parameters. Alternatively, some preparations were examined on Olympus AX-70 equipped with 40x/1.0 NA water objective. Some preparations were also examined on confocal microscope Leica SP5 with 60x/1.4 NA oil objective. Optical z-sections were acquired at 0.125 µm. Z-stacks were deconvolved by Huygens Professional software (SVI, The Netherlands). All presented Maximum Intensity Projections (MIP) of deconvolved z-stacks were prepared in ImageJ (NIH/USA). Conjugates alone gave no significant staining.

### Time-lapse imaging

For time-lapse imaging, U2OS cells expressing EB1-GFP were grown on glass-bottom-dishes (MatTek) and transfected with different sets of plasmids, as specified in Result section. Before imaging, DMEM was replaced with medium for live cell imaging (DMEM without phenol red, riboflavin, folic acid, pyridoxal, Fe[NO_3_]_3_ and puromycin). Only cells expressing TagRFP or TagRPF-fusion proteins were selected for time-lapse imaging.

Time-lapse sequences of EB1-GFP dynamics were collected for 2 min at 1 sec intervals on Delta Vision Core system (Applied Precision) equipped with 60x/1.42 NA oil-immersion objective. The focus plane was near the coverslip where the best resolution of EB1 comets was observed. Time-lapse sequences were adjusted in ImageJ (NIH, USA) by manual cropping of individual cells, enhancement of brightness and contrast, and converting sequences to a depth of 8 bits. Adjusted time-lapse sequences of individual cells were analyzed by in-house-written particle tracking plug-in implemented in Ellipse program version 2.07 (ViDiTo, Systems, Košice Slovakia; http://www.ellipse.sk) as described [42]. The particle speed was calculated as the ratio of particle trajectory length and trajectory duration. Statistical analysis was performed with the Student's two-tailed unpaired t-test using Microsoft Excel.

## Supporting Information

Figure S1 **Exogenous γ-tubulin 2 locates to centrosomes.** Human U2OS cells expressing FLAG-tagged mouse γ-tubulin 1 (a–d, Tubg1-FLAG), mouse γ-tubulin 2 (e–h, Tubg2-FLAG), human γ-tubulin 1 (i–l, TUBG1-FLAG) and human γ-tubulin 2 (m–p, TUBG2-FLAG) were stained for FLAG (red) and pericentrin (green). DNA was stained with DAPI (blue). Final images were made by maximum intensity projection of 3 deconvolved z-sections spaced at 0.25 µm. Scale bar 10 µm.
(TIF)

Figure S2 **Coimmunoprecipitation of human γ-tubulins with GCP2 and GCP4 proteins.** Extracts from HEK cells expressing FLAG-tagged human γ-tubulin 1 (TUBG1-FLAG), human γ-tubulin 2 (TUBG2-FLAG) or control mouse Fyn (Fyn-FLAG) were immunoprecipitated with antibodies to FLAG or GCP2, and blots were probed with antibodies to FLAG, GCP2, GCP4 and γ-tubulin (γ-Tb). Extracts (1), immunoprecipitated proteins (2), protein A without antibodies incubated with extracts (3), immobilized antibodies not incubated with extracts (4). Arrowheads indicate the positions of exogenous γ-tubulins.
(TIF)

Figure S3 **Immunoblot analysis of U2OS cells in phenotypic rescue experiments with FLAG-tagged γ-tubulins.** (A) Immunoblot analysis of whole cell extracts from cells transfected with negative control (Control) or γ-tubulin specific siRNAs (KD1 and KD2). Staining with antibodies to γ-tubulin (γ-Tb) and GAPDH. (B) Cells with depleted γ-tubulin 1 (KD2), expressing FLAG-tagged mouse γ-tubulin 1 (Tubg1-FLAG), mouse γ-tubulin 2 (Tubg2-FLAG) or human γ-tubulin 2 (TUBG2-FLAG). Immunoblots of whole cell lysates probed with antibodies to γ-tubulin (γ-Tb), FLAG and GAPDH (loading control). Arrowhead indicates the position of endogenous γ-tubulin.
(TIF)

Figure S4 **γ-Tubulin 2 rescues mitotic spindle organization and function in γ-tubulin 1-depleted cells.** U2OS cells depleted of γ-tubulin 1 and expressing FLAG-tagged mouse γ-tubulin 1 (a, d; Tubg1-FLAG), mouse γ-tubulin 2 (b, e; Tubg2-FLAG) or human γ-tubulin 2 (c, f; TUBG2-FLAG). Cells were stained for FLAG (red) and β-tubulin (green). DNA was stained with DAPI (blue). Final images were made by maximum intensity projection of 30–40 deconvolved confocal z-sections spaced at 0.125 µm. Scale bars 5 µm.
(TIF)

Figure S5 **Depletion of γ-tubulin 1 in U2OS cells by shRNA.** Cells transfected with empty pLKO.1 vector (Control), TUBG1 shRNA expressing vectors p9396sh (KD1) or p120194sh (KD2). (A) Immunoblots of whole cell lysates probed with antibodies to γ-tubulin (γ-Tb) and GAPDH (loading control). (B) Immunofluorescence staining with antibody to γ-tubulin (red) and with DAPI (blue). Fluorescence images of cells stained for γ-tubulin were captured under identical conditions and processed in exactly the same manner. Scale bar 20 µm.
(TIF)

Figure S6 **Immunoblot analysis of U2OS cells in phenotypic rescue experiments with TagRFP-tagged γ-tubulins.** U2OS-EB1 cells with depleted γ-tubulin 1 (KD2; shRNA) or negative control cells (NC; pLKO.1), expressing TagRFP, tagged mouse γ-tubulin 1 (Tubg1-TagRFP) or tagged human γ-tubulin 2 (TUBG2-TagRFP). Immunoblots of whole cell lysates probed with antibodies to γ-tubulin (γ-Tb) and GAPDH (loading control). Arrowhead indicates the position of endogenous γ-tubulin.
(TIF)

Figure S7 **γ-Tubulin 2 rescues microtubule formation in γ-tubulin 1-depleted cells during interphase.** Time-lapse imaging of U2OS-EB1 cells for quantitative evaluation of microtubule (+) end dynamics. Cells with depleted γ-tubulin 1 (KD2) expressing either mouse γ-tubulin 1 (pmTubg1-TagRFP) or human γ-tubulin 2 (phTUBG2-TagRFP). Single frame coloured images Fig 5c and Fig. 5d were separated to red and green channels for a better evaluation of γ-tubulin-TagRFP fusions (red) and EB1-GFP (green). White arrows mark MTOCs.
(TIF)

Figure S8 **Comparison of γ-tubulin 2 expression in mouse brain and cell lines.** Expression of gene for γ-tubulin 2 (Tubg2) in neuroblastoma (Neuro2a), bone marrow mast cells (BMMC), embryonal fibroblasts (3T3) and embryonic carcinoma cells (P19) relative to the level in brain. Data are presented as mean fold change (columns) with individual samples displayed (diamonds). Three biological replicates were quantified twice under identical conditions. *, undetectable level in P19 cells.
(TIF)

Table S1 **Sequence alignments of human and mouse γ-tubulins.**
(PDF)

Table S2 **Multiple sequence alignment of carboxy-terminal domains of mammalian γ-tubulins.**
(PDF)

Table S3 **Sequences of primers used for RT-qPCR analysis of mouse genes.**
(PDF)

Text S1 **Thermocycling parameters at quantitative PCR.**
(PDF)

## References

[1] Oakley BR, Oakley CE, Yoon Y, Jung M (1990). γ-Tubulin is a component of the spindle pole body that is essential for microtubule function in *Aspergillus nidulans*.. Cell.

[2] Stearns T, Evans L, Kirschner M (1991). γ-Tubulin is highly conserved component of the centrosome.. Cell.

[3] Joshi HC, Palacios MJ, McNamara L, Cleveland DW (1992). γ-Tubulin is a centrosomal protein required for cell cycle-dependent microtubule nucleation.. Nature.

[4] Wiese C, Zheng Y (2006). Microtubule nucleation: gamma-tubulin and beyond.. J Cell Sci.

[5] Raynaud-Messina B, Merdes A (2007). γ-Tubulin complexes and microtubule organization.. Curr Opin Cell Biol.

[6] Moritz M, Braunfeld MB, Guenebaut V, Heuser J, Agard DA (2000). Structure of the γ-tubulin ring complex: a template for microtubule nucleation.. Nat Cell Biol.

[7] Kollman JM, Polka JK, Zelter A, Davis TN, Agard DA (2010). Microtubule nucleating γ-TuSC assembles structures with 13-fold microtubule-like symmetry.. Nature.

[8] Lüders J, Stearns T (2007). Microtubule-organizing centres: a re-evaluation.. Nat Rev Mol Cell Biol.

[9] Hořejší B, Vinopal S, Sládková V, Dráberová E, Sulimenko V (2011). Nuclear γ-tubulin associates with nucleoli and interacts with tumor suppressor protein C53.. J Cell Physiol.

[10] Moudjou M, Bordes N, Paintrand M, Bornens M (1996). γ-Tubulin in mammalian cells: the centrosomal and the cytosolic forms.. J Cell Sci.

[11] Haren L, Remy MH, Bazin I, Callebaut I, Wright M (2006). NEDD1-dependent recruitment of the γ-tubulin ring complex to the centrosome is necessary for centriole duplication and spindle assembly.. J Cell Biol.

[12] Dammermann A, Maddox PS, Desai A, Oegema K (2008). SAS-4 is recruited to a dynamic structure in newly forming centrioles that is stabilized by the gamma-tubulin-mediated addition of centriolar microtubules.. J Cell Biol.

[13] Zimmerman S, Chang F (2005). Effects of γ-tubulin complex proteins on microtubule nucleation and catastrophe in fission yeast.. Mol Biol Cell.

[14] Cuschieri L, Miller R, Vogel J (2006). Gamma-tubulin is required for proper recruitment and assembly of Kar9-Bim1 complexes in budding yeast.. Mol Biol Cell.

[15] Bouissou A, Verollet C, Sousa A, Sampaio P, Wright M (2009). γ-Tubulin ring complexes regulate microtubule plus end dynamics.. J Cell Biol.

[16] Nayak T, Edgerton-Morgan H, Horio T, Xiong Y, De Souza CP (2010). Gamma-tubulin regulates the anaphase-promoting complex/cyclosome during interphase.. J Cell Biol.

[17] Rodriguez AS, Batac J, Killilea AN, Filopei J, Simeonov DR (2008). Protein complexes at the microtubule organizing center regulate bipolar spindle assembly.. Cell Cycle.

[18] Liu B, Joshi HC, Wilson TJ, Silflow CD, Palevitz BA (1994). γ-Tubulin in Arabidopsis: gene sequence, immunoblot, and immunofluorescence studies.. Plant Cell.

[19] Ruiz F, Beisson J, Rossier J, Dupuis-Williams P (1999). Basal body duplication in Paramecium requires gamma-tubulin.. Current Biology.

[20] Tan M, Heckmann K (1998). The two gamma-tubulin-encoding genes of the ciliate Euplotes crassus differ in their sequences, codon usage, transcription initiation sites and poly(A) addition sites.. Gene.

[21] Wilson PG, Zheng Y, Oakley CE, Oakley BR, Borisy GG (1997). Differential expression of two γ-tubulin isoforms during gametogenesis and development in *Drosophila*.. Dev Biol.

[22] Wise DO, Krahe R, Oakley BR (2000). The γ-tubulin gene family in humans.. Genomics.

[23] Yuba-Kubo A, Kubo A, Hata M, Tsukita S (2005). Gene knockout analysis of two γ-tubulin isoforms in mice.. Develop Biol.

[24] Carson AR, Scherer SW (2009). Identifying concerted evolution and gene conversion in mammalian gene pairs lasting over 100 million years.. BMC Evol Biol.

[25] Khodjakov A, Rieder CL (1999). The sudden recruitment of γ-tubulin to the centrosome at the onset of mitosis and its dynamic exchange throughout the cell cycle, do not require microtubules.. J Cell Biol.

[26] Piehl M, Tulu US, Wadsworth P, Cassimeris L (2004). Centrosome maturation: Measurement of microtubule nucleation throughout the cell cycle by using GFP-tagged EB1.. Proc Natl Acad Sci USA.

[27] Piko L, Clegg KB (1982). Quantitative changes in total RNA, total poly(A), and ribosomes in early mouse embryos.. Dev Biol.

[28] Schultz RM, Wassarman PM (1977). Biochemical studies of mammalian oogenesis: Protein synthesis during oocyte growth and meiotic maturation in the mouse.. J Cell Sci.

[29] Sellens MH, Stein S, Sherman MI (1981). Protein and free amino acid content in preimplantation mouse embryos and in blastocysts under various culture conditions.. J Reprod Fertil.

[30] Guillet V, Knibiehler M, Gregory-Pauron L, Remy MH, Chemin C (2011). Crystal structure of gamma-tubulin complex protein GCP4 provides insight into microtubule nucleation.. Nat Struct Mol Biol.

[31] Lüders J, Patel UK, Stearns T (2006). GCP-WD is a γ-tubulin targeting factor required for centrosomal and chromatin mediated microtubule nucleation.. Nature Cell Biol.

[32] Uehara R, Nozawa RS, Tomioka A, Petry S, Vale RD (2009). The augmin complex plays a critical role in spindle microtubule generation for mitotic progression and cytokinesis in human cells.. Proc Natl Acad Sci U S A.

[33] Uehara R, Goshima G (2010). Functional central spindle assembly requires de novo microtubule generation in the interchromosomal region during anaphase.. J Cell Biol.

[34] Hutchins JR, Toyoda Y, Hegemann B, Poser I, Heriche JK (2010). Systematic analysis of human protein complexes identifies chromosome segregation proteins.. Science.

[35] Hendrickson TW, Yao J, Bhadury S, Corbett AH, Joshi HC (2001). Conditional mutations in gamma-tubulin reveal its involvement in chromosome segregation and cytokinesis.. Mol Biol Cell.

[36] Lynch M, Force A (2000). The probability of duplicate gene preservation by subfunctionalization.. Genetics.

[37] Lam EW, Glassford J, Banerji L, Thomas NS, Sicinski P (2000). Cyclin D3 compensates for loss of cyclin D2 in mouse B-lymphocytes activated via the antigen receptor and CD40.. J Biol Chem.

[38] Kafri R, Levy M, Pilpel Y (2006). The regulatory utilization of genetic redundancy through responsive backup circuits.. Proc Natl Acad Sci U S A.

[39] DeLuna A, Springer M, KIrschner MW, Kishony R (2010). Need-based up-regulation of protein levels in response to deletion of their duplicate genes.. PLoS Biol.

[40] Katsetos CD, Reddy G, Dráberová E, Šmejkalová B, Del Valle L (2006). Altered cellular distribution and subcellular sorting of γ-tubulin in diffuse astrocytic gliomas and human glioblastoma cell lines.. J Neuropathol Exp Neurol.

[41] Loh JK, Lieu AS, Chou CH, Lin FY, Wu CH (2010). Differential expression of centrosomal proteins at different stages of human glioma.. Bmc Cancer.

[42] Hájková Z, Bugajev V, Dráberová E, Vinopal S, Dráberová L (2011). STIM1-directed reorganization of microtubules in activated cells.. J Immunol.

[43] Mimori-Kiyosue Y, Shiina N, Tsukita S (2004). The dynamic behavior of the APC-binding protein EB1 on the distal ends of microtubules.. Curr Biol.

[44] Nováková M, Dráberová E, Schürmann W, Czihak G, Viklický V (1996). γ-Tubulin redistribution in taxol-treated mitotic cells probed by monoclonal antibodies.. Cell Motil Cytoskel.

[45] Dráberová E, Sulimenko V, Kukharskyy V, Dráber P (1999). Monoclonal antibody NF-09 specific for neurofilament protein NF-M.. Folia Biol (Prague).

[46] Laemmli UK (1970). Cleavage of structural proteins during the assembly of the head of bacteriophage T_4_.. Nature.

[47] Sulimenko V, Sulimenko T, Poznanovic S, Nechiporuk-Zloy V, Böhm JK (2002). Association of brain γ-tubulins with αβ-tubulin dimers.. Biochem J.

[48] Dráber P (1991). Quantitation of proteins in sample buffer for sodium dodecyl sulfate-polyacrylamide gel electrophoresis using colloidal silver.. Electrophoresis.

[49] Kukharskyy V, Sulimenko V, Macůrek L, Sulimenko T, Dráberová E (2004). Complexes of γ-tubulin with non-receptor protein tyrosine kinases Src and Fyn in differentiating P19 embryonal carcinoma cells.. Exp Cell Res.

[50] Dráberová E, Dráber P (1993). A microtubule-interacting protein involved in coalignment of vimentin intermediate filaments with microtubules.. J Cell Sci.

