# Correction: γ-Tubulin 2 Nucleates Microtubules and Is Downregulated in Mouse Early Embryogenesis

**DOI:** 10.1371/annotation/5dd084b1-20e6-4e1f-88e0-dfe05289da08

**Published:** 2012-06-28

**Authors:** Stanislav Vinopal, Markéta Černohorská, Vadym Sulimenko, Tetyana Sulimenko, Věra Vosecká, Matyáš Flemr, Eduarda Dráberová, Pavel Dráber

There is an error in the Funding. Grant P302/10/1759(ED) is incorrect. The correct number is P302/10/1701(ED). 

