# Supplementary figures and images for: γ-Tubulin 2 Nucleates Microtubules and Is Downregulated in Mouse Early Embryogenesis
Source: PLoS One. 2012 Jan 3;7(1):e29919. doi: 10.1371/journal.pone.0029919 (PMC3250491; doi:10.1371/journal.pone.0029919)

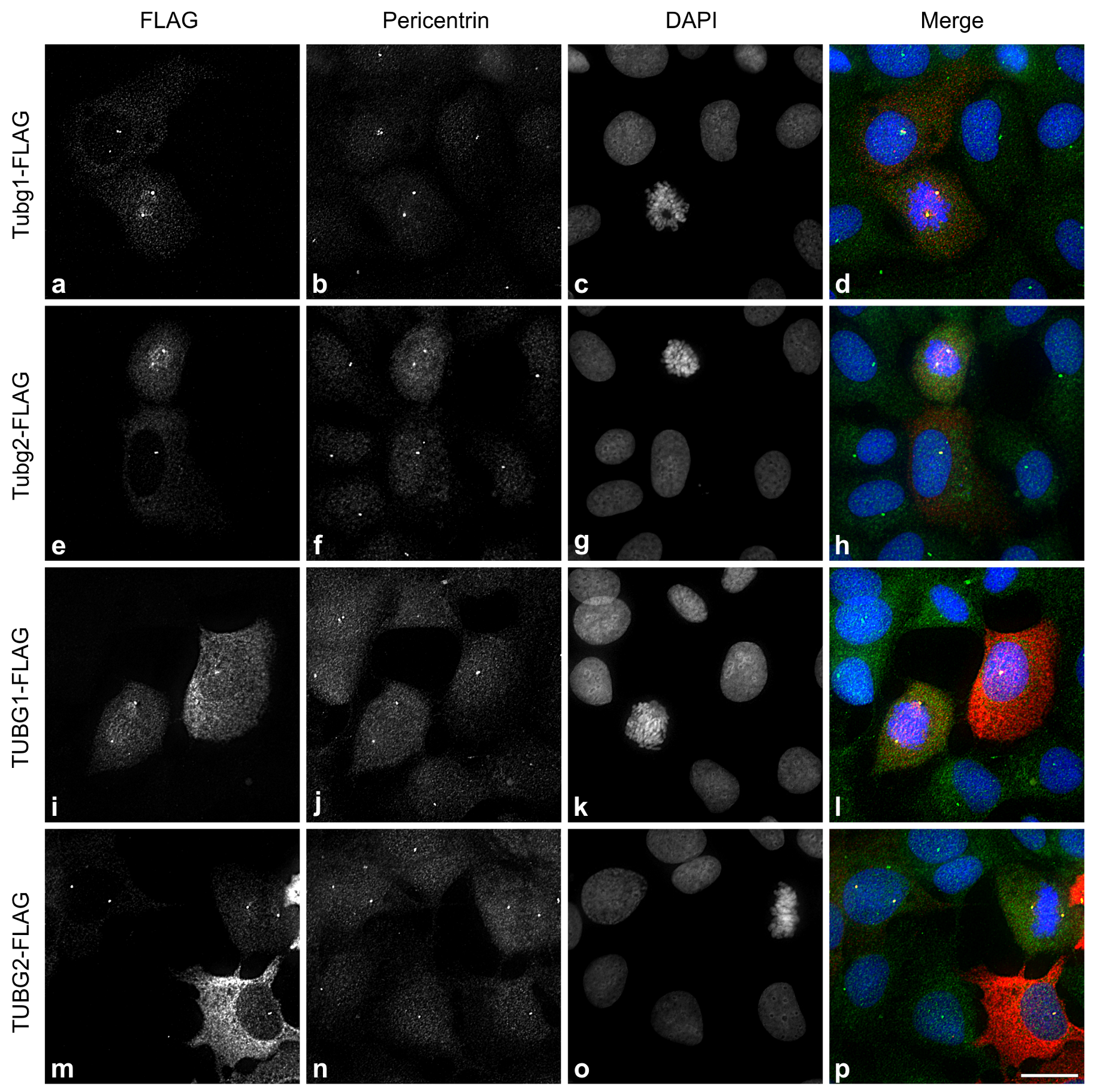

Supplement: Figure S1 — Exogenous γ-tubulin 2 locates to centrosomes. Human U2OS cells expressing FLAG-tagged mouse γ-tubulin 1 (a–d, Tubg1-FLAG), mouse γ-tubulin 2 (e–h, Tubg2-FLAG), human γ-tubulin 1 (i–l, TUBG1-FLAG) and human γ-tubulin 2 (m–p, TUBG2-FLAG) were stained for FLAG (red) and pericentrin (green). DNA was stained with DAPI (blue). Final images were made by maximum intensity projection of 3 deconvolved z-sections spaced at 0.25 µm. Scale bar 10 µm. (TIF) [file pone.0029919.s001.tif]

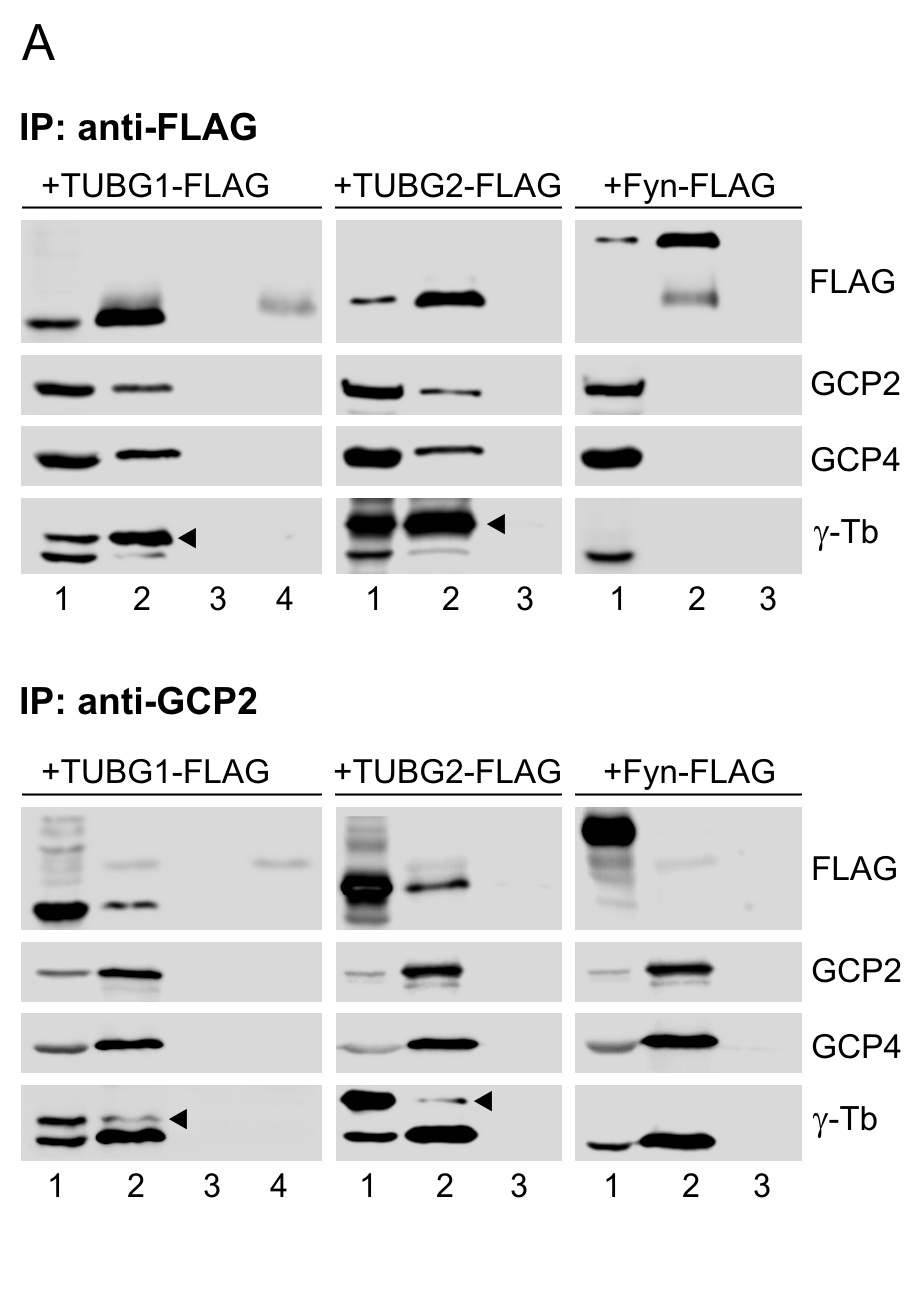

Supplement: Figure S2 — Coimmunoprecipitation of human γ-tubulins with GCP2 and GCP4 proteins. Extracts from HEK cells expressing FLAG-tagged human γ-tubulin 1 (TUBG1-FLAG), human γ-tubulin 2 (TUBG2-FLAG) or control mouse Fyn (Fyn-FLAG) were immunoprecipitated with antibodies to FLAG or GCP2, and blots were probed with antibodies to FLAG, GCP2, GCP4 and γ-tubulin (γ-Tb). Extracts (1), immunoprecipitated proteins (2), protein A without antibodies incubated with extracts (3), immobilized antibodies not incubated with extracts (4). Arrowheads indicate the positions of exogenous γ-tubulins. (TIF) [file pone.0029919.s002.tif]

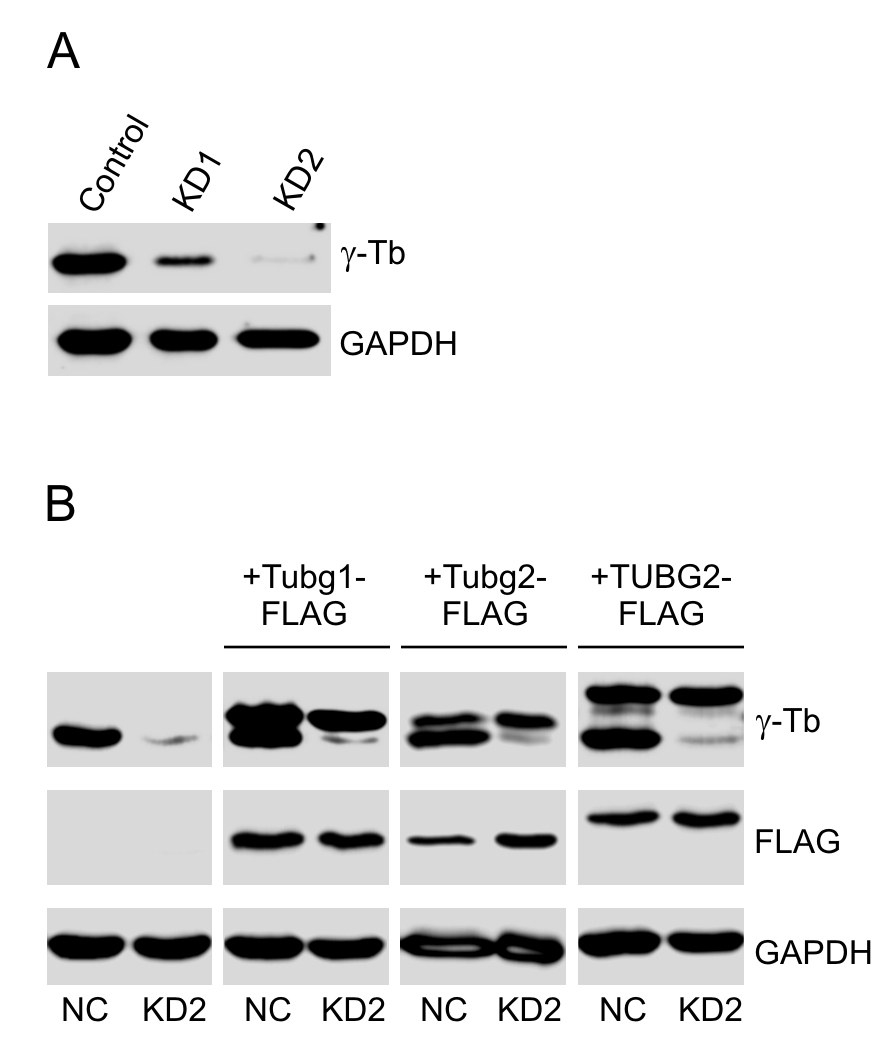

Supplement: Figure S3 — Immunoblot analysis of U2OS cells in phenotypic rescue experiments with FLAG-tagged γ-tubulins. (A) Immunoblot analysis of whole cell extracts from cells transfected with negative control (Control) or γ-tubulin specific siRNAs (KD1 and KD2). Staining with antibodies to γ-tubulin (γ-Tb) and GAPDH. (B) Cells with depleted γ-tubulin 1 (KD2), expressing FLAG-tagged mouse γ-tubulin 1 (Tubg1-FLAG), mouse γ-tubulin 2 (Tubg2-FLAG) or human γ-tubulin 2 (TUBG2-FLAG). Immunoblots of whole cell lysates probed with antibodies to γ-tubulin (γ-Tb), FLAG and GAPDH (loading control). Arrowhead indicates the position of endogenous γ-tubulin. (TIF) [file pone.0029919.s003.tif]

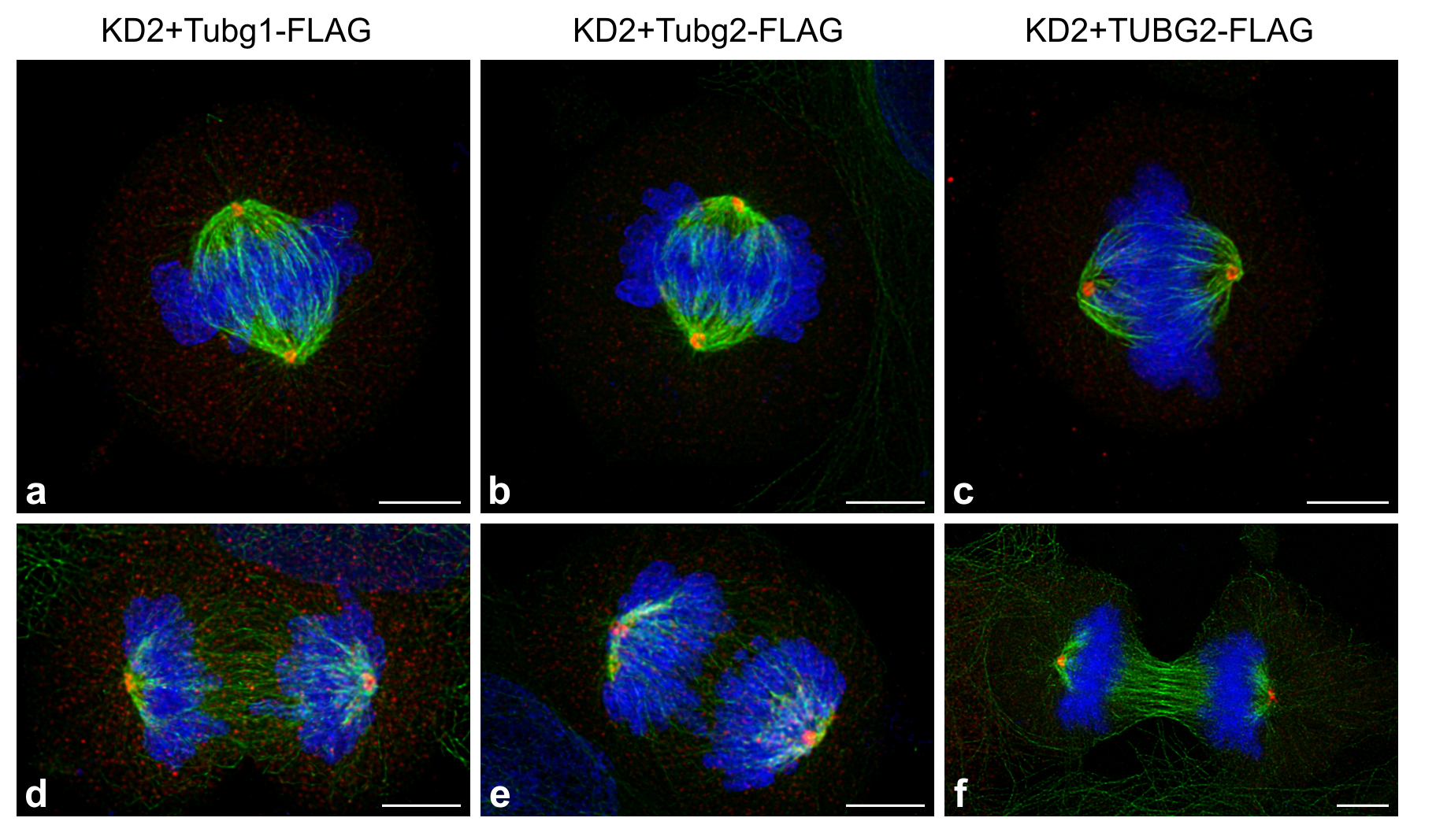

Supplement: Figure S4 — γ-Tubulin 2 rescues mitotic spindle organization and function in γ-tubulin 1-depleted cells. U2OS cells depleted of γ-tubulin 1 and expressing FLAG-tagged mouse γ-tubulin 1 (a, d; Tubg1-FLAG), mouse γ-tubulin 2 (b, e; Tubg2-FLAG) or human γ-tubulin 2 (c, f; TUBG2-FLAG). Cells were stained for FLAG (red) and β-tubulin (green). DNA was stained with DAPI (blue). Final images were made by maximum intensity projection of 30–40 deconvolved confocal z-sections spaced at 0.125 µm. Scale bars 5 µm. (TIF) [file pone.0029919.s004.tif]

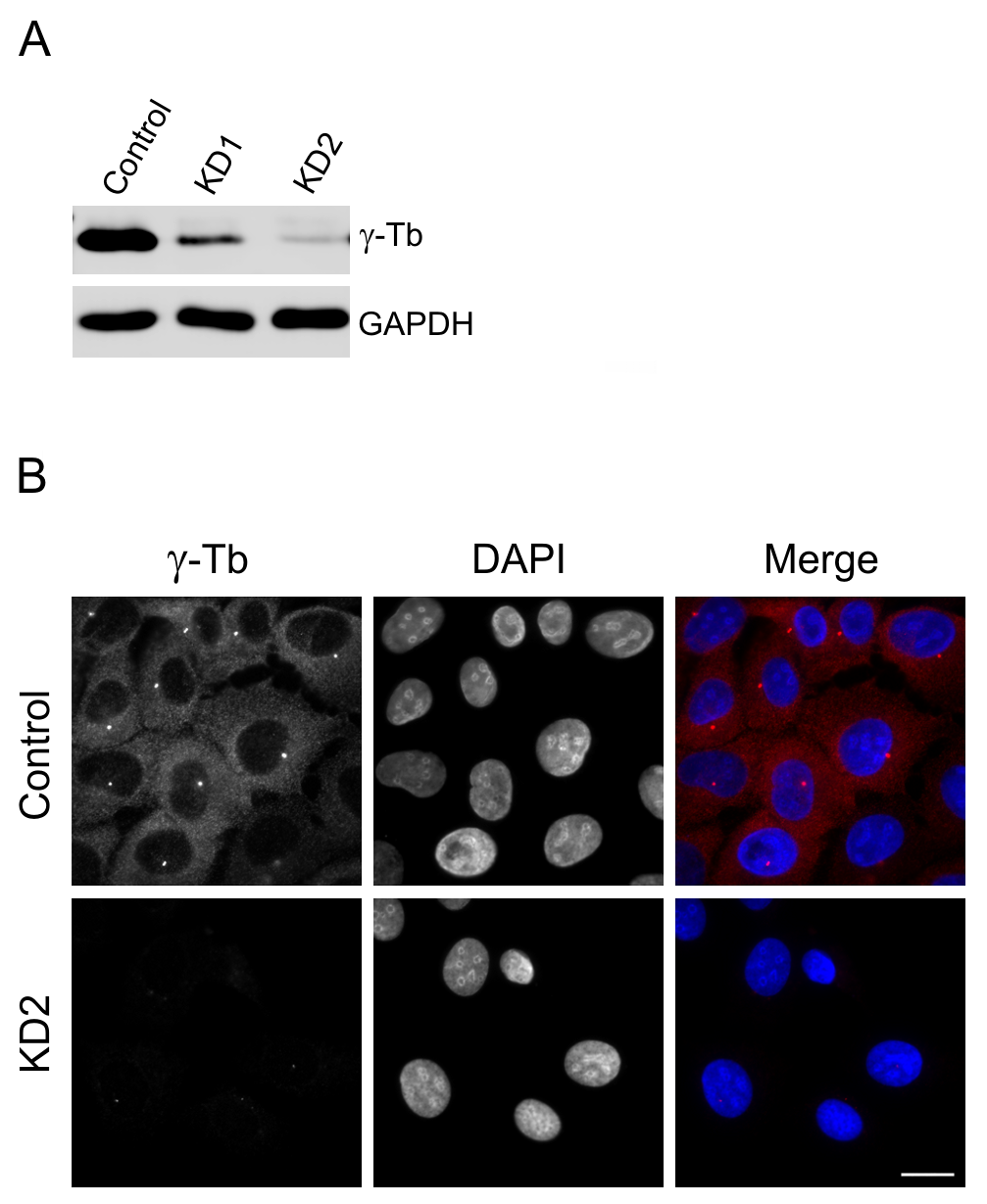

Supplement: Figure S5 — Depletion of γ-tubulin 1 in U2OS cells by shRNA. Cells transfected with empty pLKO.1 vector (Control), TUBG1 shRNA expressing vectors p9396sh (KD1) or p120194sh (KD2). (A) Immunoblots of whole cell lysates probed with antibodies to γ-tubulin (γ-Tb) and GAPDH (loading control). (B) Immunofluorescence staining with antibody to γ-tubulin (red) and with DAPI (blue). Fluorescence images of cells stained for γ-tubulin were captured under identical conditions and processed in exactly the same manner. Scale bar 20 µm. (TIF) [file pone.0029919.s005.tif]

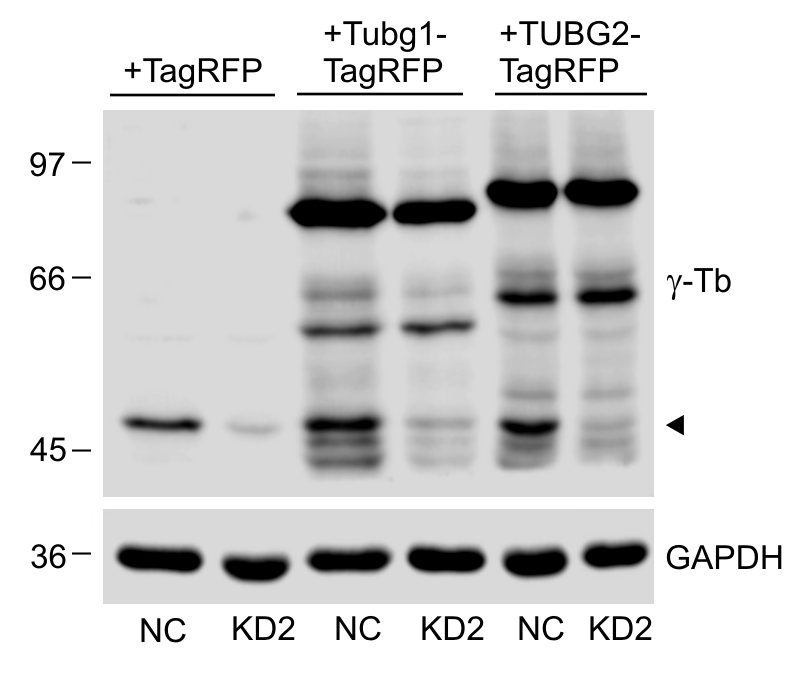

Supplement: Figure S6 — Immunoblot analysis of U2OS cells in phenotypic rescue experiments with TagRFP-tagged γ-tubulins. U2OS-EB1 cells with depleted γ-tubulin 1 (KD2; shRNA) or negative control cells (NC; pLKO.1), expressing TagRFP, tagged mouse γ-tubulin 1 (Tubg1-TagRFP) or tagged human γ-tubulin 2 (TUBG2-TagRFP). Immunoblots of whole cell lysates probed with antibodies to γ-tubulin (γ-Tb) and GAPDH (loading control). Arrowhead indicates the position of endogenous γ-tubulin. (TIF) [file pone.0029919.s006.tif]

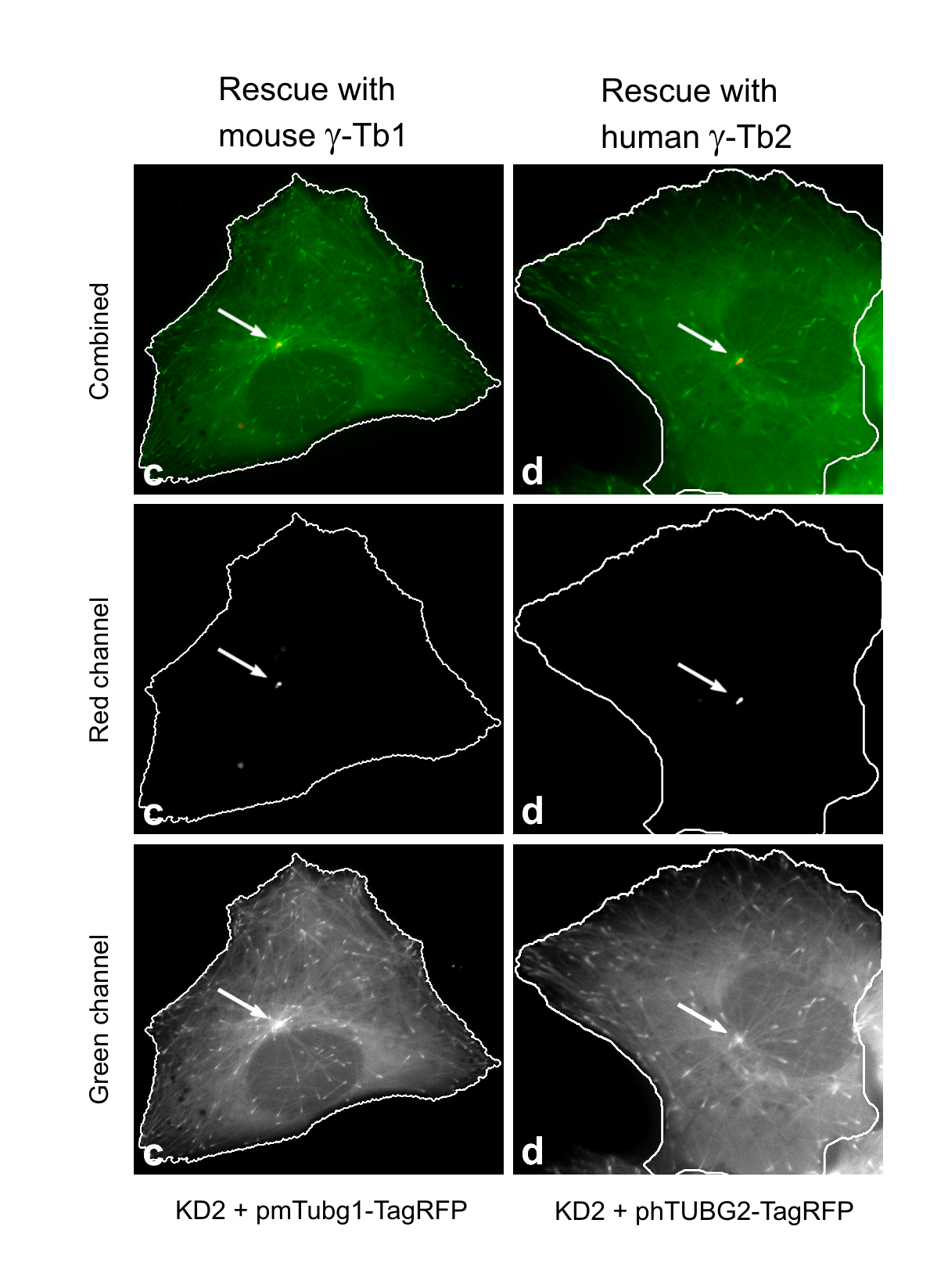

Supplement: Figure S7 — γ-Tubulin 2 rescues microtubule formation in γ-tubulin 1-depleted cells during interphase. Time-lapse imaging of U2OS-EB1 cells for quantitative evaluation of microtubule (+) end dynamics. Cells with depleted γ-tubulin 1 (KD2) expressing either mouse γ-tubulin 1 (pmTubg1-TagRFP) or human γ-tubulin 2 (phTUBG2-TagRFP). Single frame coloured images Fig 5c and Fig. 5d were separated to red and green channels for a better evaluation of γ-tubulin-TagRFP fusions (red) and EB1-GFP (green). White arrows mark MTOCs. (TIF) [file pone.0029919.s007.tif]

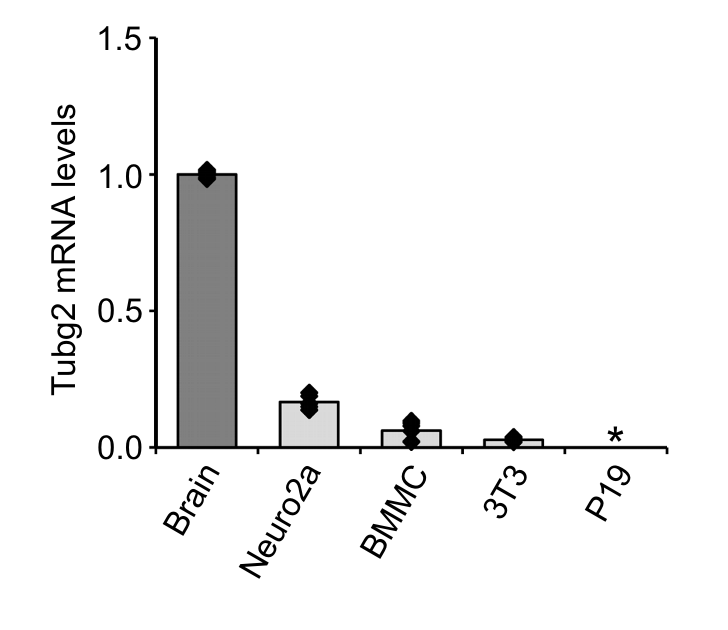

Supplement: Figure S8 — Comparison of γ-tubulin 2 expression in mouse brain and cell lines. Expression of gene for γ-tubulin 2 (Tubg2) in neuroblastoma (Neuro2a), bone marrow mast cells (BMMC), embryonal fibroblasts (3T3) and embryonic carcinoma cells (P19) relative to the level in brain. Data are presented as mean fold change (columns) with individual samples displayed (diamonds). Three biological replicates were quantified twice under identical conditions. *, undetectable level in P19 cells. (TIF) [file pone.0029919.s008.tif]
