# Supplementary material for: γ-Tubulin 2 Nucleates Microtubules and Is Downregulated in Mouse Early Embryogenesis
Source: PLoS One. 2012 Jan 3;7(1):e29919. doi: 10.1371/journal.pone.0029919 (PMC3250491; doi:10.1371/journal.pone.0029919)
Supplement: Table S1 — Sequence alignments of human and mouse γ-tubulins. (PDF) [file pone.0029919.s009.pdf]

Table S1 Alignments of human and mouse γ-tubulins

γ-Tubulin 1

```
H.s. MPREIITLQLGQCGNQIGFEFWKQLCAEHGISPEGIVEEFATEGTDRKDVFFYQADDEHYIPRAVLLDLEPRVIHSILNSPYAKLYNPENIYLSEHGGGAGNNWASGFSQGEKIHEDIFD 120
M.m. MPREIITLQLGQCGNQIGFEFWKQLCAEHGISPEGIVEEFATEGTDRKDVFFYQADDEHYIPRAVLLDLEPRVIHSILNSSYAKLYNPENIYLSEHGGGAGNNWASGFSQGEKIHEDIFD 120
     ********************************************************************************.***************************************

H.s. IIDREADGSDSLEGFVLCHSIAGGTGSGLGSYLLERLNDRYPKKLVQTYSVFPNQDEMSDVVVQPYNSLLTLKRLTQNADCVVVLDNTALNRIATDRLHIQNPSFSQINQLVSTIMSAST 240
M.m. IIDREADGSDSLEGFVLCHSIAGGTGSGLGSYLLERLNDRYPKKLVQTYSVFPNQDEMSDVVVQPYNSLLTLKRLTQNADCVVVLDNTALNLIATDRLHIQNPSFSQINQLVSTIMSAST 240
     ******************************************************************************************* ****************************

H.s. TTLRYPGYMNNDLIGLIASLIPTPRLHFLMTGYTPLTTDQSVASVRKTTVLDVMRRLLQPKNVMVSTGRDRQTNHCYIAILNIIQGEVDPTQVHKSLQRIRERKLANFIPWGPASIQVAL 360
M.m. TTLRYPGYMNNDLIGLIASLIPTPRLHFLMTGYTPLTTDQSVASVRKTTVLDVMRRLLQPKNVMVSTGRDRQTNHCYIAILNIIQGEVDPTQVHKSLQRIRERKLANFIPWGPASIQVAL 360
     ************************************************************************************************************************

H.s. SRKSPYLPSAHRVSGLMMANHTSISSLFERTCRQYDKLRKREAFLEQFRKEDMFKDNFDEMDTSREIVQQLIDEYHAATRPDYISWGTQEQ 451
M.m. SRKSPYLPSAHRVSGLMMANHTSISSLFERTCRQFDKLRKREAFMEQFRKEDIFKDNFDEMDTSREIVQQLIDEYHAATRPDYISWGTQEQ 451
     **********************************:*********:*******:**************************************
```

γ-Tubulin 2

```
H.s. MPREIITLQLGQCGNQIGFEFWKQLCAEHGISPEGIVEEFATEGTDRKDVFFYQADDEHYIPRAVLLDLEPRVIHSILNSPYAKLYNPENIYLSEHGGGAGNNWASGFSQGEKIHEDIFD 120
M.m. MPREIITLQLGQCGNQIGFEFWKQLCAEHGISPEGIVEEFATEGTDRKDVFFYQADDEHYIPRAVLLDLEPRVIHSILNSSYAKLYNPENIYLSEHGGGAGNNWGRGFSQGEKIHEDIFD 120
     ********************************************************************************.***********************. **************

H.s. IIDREADGSDSLEGFVLCHSIAGGTGSGLGSYLLERLNDRYPKKLVQTYSVFPYQDEMSDVVVQPYNSLLTLKRLTQNADCVVVLDNTALNRIATDRLHIQNPSFSQINQLVSTIMSAST 240
M.m. IIDREADGSDSLEGFVLCHSIAGGTGSGLGSYLLERLNDRYPKKLVQTYSVFPNQDEMSDVVVQPYNSLLTLKRLTQNADCVVVLDNTALNRIATDRLHIQNPSFSQINQLVSTIMSAST 240
     ***************************************************** ******************************************************************

H.s. TTLRYPGYMNNDLIGLIASLIPTPRLHFLMTGYTPLTTDQSVASVRKTTVLDVMRRLLQPKNVMVSTGRDRQTNHCYIAILNIIQGEVDPTQVHKSLQRIRERKLANFIPWGPASIQVAL 360
M.m. TTLRYPGYMNNDLIGLIASLIPTPRLHFLMTGYTPLTTDQSVASVRKTTVLDVMRRLLQPKNVMVSTGRDRQTNHCYIAILNIIQGEVDPTQVHKSLQRIRERKLANFIPWGPASIQVAL 360
     ************************************************************************************************************************

H.s. SRKSPYLPSAHRVSGLMMANHTSISSLFESSCQQFDKLRKRDAFLEQFRKEDMFKDNFDEMDRSREVVQELIDEYHAATQPDYISWGTQEQ 451
M.m. SRKSPYLPSAHRVSGLMMANHTSISSLFESSCQQYDKLWKRGAFLEQFRKEDIFKDNFEEMHRSREVVQELIDEYHAATRPDYISWGTQEQ 451
     **********************************:*** **.**********:*****:**.*****************:***********
```

H.s., human (*Homo sapiens*); *M. m.*, mouse (*Mus musculus*). Accesions numbers (NCBI database): human γ-tubulin 1, NP_001061.2; mouse γ-tubulin 1, NP_598785.1; human γ-tubulin 2, NP_057521.1; mouse γ-tubulin 2, NP_598789.1. An asterisk indicates positions which have a single, fully conserved residue. A colon indicates conservation among groups of strongly similar properties. A period indicates conservation among groups of weakly similar properties. Different amino acids are highlighted.
