# Supplementary material for: γ-Tubulin 2 Nucleates Microtubules and Is Downregulated in Mouse Early Embryogenesis
Source: PLoS One. 2012 Jan 3;7(1):e29919. doi: 10.1371/journal.pone.0029919 (PMC3250491; doi:10.1371/journal.pone.0029919)
Supplement: Table S2 — Multiple sequence alignment of carboxy-terminal domains of mammalian γ-tubulins. (PDF) [file pone.0029919.s010.pdf]

Table S2. Multiple sequence alignment of carboxy-terminal domains of mammalian γ-tubulins

Species	Part of helix H11	Part of helix H12	Accesion number
**γ-tubulin 2**			
Human (*Homo sapiens*)	(386) SLFESSCQQFDK	(419) DEMDRSREVVQELIDE	NP_057521.1
Chimpanzee (*Pan troglodytes*)	(386) SLFESSCQQFDK	(419) DEMDRSREVVQELIDE	XP_003315538
Mouse (*Mus musculus*)	(386) SLFESSCQQYDK	(419) EEMHRSREVVQELIDE	NP_598789.1
Rat (*Rattus norvegicus*)	(411) SLFESSCQQYDK	(444) EEMDRSREVVQELIDE	NP_001178004.1
Horse (*Equus caballus*)	(386) SLFESSCQQYDK	(419) DELDRSREVVQELIDE	XP_001493749.1
Bat (*Myotis lucifugus*)	(386) SLFESSCQQYDK	(419) DELDRSREVVQELIDE	ENSMLUP00000007140 (Ensembl)
Bovine (*Bos taurus*)	(386) SLFESSCQQYDK	(419) DELDRSREVVQELIDE	NP_001032704.1
Dog (*Canis familiaris*)	(386) SLFESSCQQYDK	(419) DELDTSREVVQELIDE	XP_548085.2
**γ-tubulin 1**			
Human (*Homo sapiens*)	(386) SLFERTCRQYDK	(419) DEMDTSREIVQQLIDE	NP_001061.2
Chimpanzee (*Pan troglodytes*)	(386) SLFERTCRQYDK	(419) DEMDTSREIVQQLIDE	XP_001162243
Mouse (*Mus musculus*)	(386) SLFERTCRQFDK	(419) DEMDTSREIVQQLIDE	NP_598785.1
Rat (*Rattus norvegicus*)	(386) SLFERTCRQFDK	(419) DEMDTSREIVQQLIDE	NP_665721.1
Horse (*Equus caballus*)	(386) SLFERTCRQYDK	(419) DELDTSREIVQQLIDE	XP_001493897.2
Bat (*Myotis lucifugus*)	(386) SLFERTCRQYDK	(419) DELDTSREIVQQLIDE	ENSMLUP00000015217 (Ensembl)
Bovine (*Bos taurus*)	(386) SLFERTCRQYDK	(419) DELDTSREIVQQLIDE	NP_001069723.1
Dog (*Canis familiaris*)	(386) SLFERTCRQYDK	(419) DELDTSREVVHQLIDE	NP_001003105.1
	**** :*:*:**	:*:. ***:*::****	

Protein sequences of indicated mammalian γ-tubulins were aligned by online version of ClustalW2 (www.ebi.ac.uk/Tools/msa/clustalw2) using default parametres. Only clusters of conserved amino acids located on helix H11 and helix H12 of γ-tubulin are shown. Numbers in brackets indicate the position of the first amino acid in the whole polypeptide chain. Accesion numbers to NCBI or Ensembl databases are provided. An asterisk indicates positions which have one single, fully conserved residue. A colon indicates conservation among groups of strongly similar properties. A period indicates conservation among groups of weakly similar properties. Conserved γ-tubulin 2-specific amino acids are highlighted.
