# Supplementary material for: γ-Tubulin 2 Nucleates Microtubules and Is Downregulated in Mouse Early Embryogenesis
Source: PLoS One. 2012 Jan 3;7(1):e29919. doi: 10.1371/journal.pone.0029919 (PMC3250491; doi:10.1371/journal.pone.0029919)
Supplement: Table S3 — Sequences of primers used for RT-qPCR analysis of mouse genes. (PDF) [file pone.0029919.s011.pdf]

Table S3. Sequences of primers used for RT-qPCR analysis of mouse genes

Name	Sequence	Amplicon length
Tubg1, fwd	5’-GCCACACGGCCAGACTATAT-3’	131 bp
Tubg1, rev	5’-TCCCTGATCTGTGCTCCGAG-3’	
Tubg2, fwd	5’-GAGGAGATGCACAGATCGAGG-3’	140 bp
Tubg2, rev	5’-GGACTGTGCTTCTTGTCCAGG-3’	
Tubgcp2, fwd	5’-CGGGCTTGAGCTCAGACACAGTTT-3’	220 bp
Tubgcp2, rev	5’-GGCGGCCACAGGGAGGATTC-3’	
Tubgcp5, fwd	5’-TGTCGTCCGGGCATCTCACCT-3’	185 bp
Tubgcp5, rev	5’-TCCCTTGCGCCGTCCCATAG-3’	
Ppia, fwd	5’-CGCGTCTCCTTCGAGCTGTTTG-3’	150 bp
Ppia, rev	5’-TGTAAAGTCACCACCCTGGCACAT-3’	
Gadph, fwd	5’-AACTTTGGCATTGTGGAAGG-3’	68 bp
Gapdh, rev	5’-ATCCACAGTCTTCTGGGTGG-3’	
