# Supplementary material for: γ-Tubulin 2 Nucleates Microtubules and Is Downregulated in Mouse Early Embryogenesis
Source: PLoS One. 2012 Jan 3;7(1):e29919. doi: 10.1371/journal.pone.0029919 (PMC3250491; doi:10.1371/journal.pone.0029919)
Supplement: Text S1 — Thermocycling parameters at quantitative PCR. (PDF) [file pone.0029919.s012.pdf]

Text S1 Thermocycling parameters at quantitative PCR

Data presented in Fig. 7 and Fig. S7 were obtained in the LightCycler® 480 (Roche) using the following thermocycling parameters: initial denaturation - 95°C/5 min; cycling - 45 cycles.

Target temperature	Fluorescence acquisition mode	Hold	Ramp Rate
95°C	None	10 s	4.8°C/s
60°C	None	20 s	2.5°C/s
72°C	Single	20 s	4.8°C/s

Melting curve

Target temperature	Fluorescence acquisition mode	Hold	Ramp Rate	Acquisitions #
95°C	None	10 s	4.8°C/s	X
60°C	None	20 s	2.5°C/s	X
72°C	Continuous	X	0.06°C/s	10/1°C
40°C	None	40 s	2.5°C/s	X

Cp values of all samples were determined in LightCycler® 480 Software, release 1.5.0, by the module “Abs quant/2nd Derivative Max”. Melting curves were analyzed in the module ”Melting curve genotyping”. Only samples with the correct melting and amplification curves were further evaluated. PCR efficiencies (E) for probed genes were calculated from calibration curves by the LightCycler® 480 Software. Calculation of the normalized relative quantity (NRQ) of evaluated transcripts was based on the following formula [1]:

$$NRQ = \frac{E_{goi}^{\Delta Cp,goi}}{\sqrt[f]{\prod_{0}^{f} E_{ref_0}^{\Delta Cp,ref_0}}}$$

taking into account different PCR efficiencies of the gene of interest (goi) and multiple reference genes (ref) used for normalization.

Expression of mouse hypoxanthine guanine phosphoribosyl transferase (*Hprt*, NM_013556), mouse peptidylprolyl isomerase A (*Ppia*, NM_008907) and mouse glyceraldehyde-3-phosphate dehydrogenase (*Gapdh*, NM_008084) was determined and their average expression stability (M) calculated as described previously [2]. *Hprt* and *Ppia* are considered to be suitable house-keeping genes during mouse early embryogenesis [3]. However, *Hpr*t was excluded from internal control gene set, because it was the least stable gene in the analyzed sample collection. Thus, geometric mean of *Ppia* and *Gapdh* expression was used for normalization.

References

1. Hellemans J, Mortier G, De Paepe A, Speleman F, Vandesompele J (2007) qBase relative quantification framework and software for management and automated analysis of real-time quantitative PCR data. Genome Biol 8: R19.

2. Vandesompele J, De Preter K, Pattyn F, Poppe B, Van Roy N et al. (2002) Accurate normalization of real-time quantitative RT-PCR data by geometric averaging of multiple internal control genes. Genome Biol 3: RESEARCH0034.

3. Mamo S, Gal AB, Bodo S, Dinnyes A (2007) Quantitative evaluation and selection of reference genes in mouse oocytes and embryos cultured in vivo and in vitro. BMC Dev Biol 7: 14.
